# Promoter Strength Driving TetR Determines the Regulatory Properties of Tet-Controlled Expression Systems

**DOI:** 10.1371/journal.pone.0041620

**Published:** 2012-07-27

**Authors:** Christiane Georgi, Julia Buerger, Wolfgang Hillen, Christian Berens

**Affiliations:** Lehrstuhl für Mikrobiologie, Department Biologie, Friedrich-Alexander-Universität Erlangen-Nürnberg, Erlangen, Germany; Institut National de la Recherche Agronomique, France

## Abstract

Bacteria frequently rely on transcription repressors and activators to alter gene expression patterns in response to changes in the surrounding environment. Tet repressor (TetR) is a paradigm transcription factor that senses the environmental state by binding small molecule effectors, the tetracyclines. However, recently isolated peptides that act as inducers of TetR after having been fused to the C-terminus of a carrier protein, suggest that TetR can also regulate gene expression in a signal-transduction pathway. For this shift in regulatory mechanism to be successful, induction of TetR must be sensitive enough to respond to an inducing protein expressed at its endogenous level. To determine this regulatory parameter, a synthetic Tet-regulated system was introduced into the human pathogen *Salmonella enterica* serovar Typhimurium and tested for inducibility by a peptide. Reporter gene expression was detected if the peptide-containing carrier protein Thioredoxin 1 was strongly overproduced, but not if it was expressed at a level similar to the physiological level of Thioredoxin 1. This was attributed to high steady-state amounts of TetR which was expressed by the promoter of the chloramphenicol acetyl transferase gene (P_cat_). Reducing P_cat_ strength either by directed or by random mutagenesis of its -10 element concomitantly reduced the intracellular amounts of TetR. Sensitive and quantitative induction of TetR by an inducing peptide, when it was fused to Thioredoxin 1 at its native locus in the genome, was only obtained with weak P_cat_ promoter variants containing GC-rich -10 elements. A second important observation was that reducing the TetR steady-state level did not impair repression. This permits flexible adjustment of an inducible system’s sensitivity simply by altering the expression level of the transcription factor. These two new layers of expression control will improve the quality and, thus, the applicability of the Tet and other regulatory systems.

## Introduction

Survival and proliferation of bacteria depend on their expressing the right amounts of the right genes at the right time. However, what is “right” at any given time-point will vary with the environmental conditions and the specific growth phase. Bacteria often respond to these changing environmental stimuli by switching the expression of specific genes “on” or “off”. To ensure that target gene expression is optimal, will require fine-tuning of the regulatory parameters that control the switch, and this fine-tuning can affect each individual step of gene expression. In bacteria, gene expression is frequently controlled by proteins that activate or repress transcription by binding to specific DNA sequences close to a promoter [1]. The DNA binding activity of these transcription factors is triggered by small molecules or, less often, by protein-protein interactions.

Tet repressor (TetR) is a paradigm for a bacterial transcription factor that responds directly to an environmental signal by binding a small molecule [2], [3]. TetR regulates transcription of the resistance protein TetA in at least 14 different efflux-type tetracycline resistance determinants found predominantly in Gram-negative bacteria [4]. Repression by TetR has to be tight, because overproduction or constitutive expression of the membrane transporter TetA strongly reduces bacterial fitness [5], [6]. But, at the same time, induction must be sensitive to ensure that TetA is translated before the antibiotic reaches an intracellular level that inhibits translation [7]. Although seemingly conflicting, these requirements are met by the exceptionally high specificity of TetR for its cognate binding site *tetO* over non-specific DNA [8] and by its unusually high affinity for tetracyclines [8], [9]. Such favorable properties have made TetR a very popular tool for many different applications, including conditional gene expression in both pro- [10] and eukaryotes [11], [12], overexpression of heterologous proteins [13] or artificial genetic circuits in synthetic biology with highly diverse architectures [14]–[18].

So far, all applications using Tet regulation have relied on tetracycline or its analogs as inducers. The recent discovery that peptides can also specifically induce TetR when they are fused to a carrier protein [19]–[21] added a new quality to Tet regulation. These inducing peptides, called TIP (TetR-inducing peptide), bind to the tetracycline-binding pocket of TetR and elicit an allosteric conformational change that leads to the complete loss of DNA-binding activity [22], [23]. This turned TetR from an exclusively small-molecule-controlled protein into a downstream effector in a protein signal transduction pathway. Examples of protein-induced regulation of gene expression are not so common in bacteria, but have been found among the major transcription factor families [24]–[26]. Information transfer by protein-mediated signal transduction not only introduces new ways to manipulate TetR-based genetic networks in synthetic biology. It also allows to gather proteomic data by determining protein expression profiles after tagging many different proteins with TIP and monitoring their expression by genetic readout of the TetR-controlled reporter gene [19], [27].

Compared with the intensely studied and well-characterized induction of gene expression by tetracyclines, the parameters for sensitive and efficient control of a Tet-regulated reporter gene by a protein-based inducer are still largely unknown. While the basic functionality of TIP-mediated induction of TetR has been demonstrated in *Escherichia coli* [19], [27] and in *Staphylococcus aureus* [28], major obstacles must still be overcome, if this system is to be used effectively in more sophisticated applications, like those mentioned above. In the examples published so far, efficient induction of TetR was only achieved after strong overproduction of the TIP-containing fusion protein from a multicopy plasmid [19], [28]. In agreement with this result, if the TIP coding sequence was fused to a gene at its native locus in the genome, induction of TetR by the resulting fusion protein was rather inefficient, because reporter activity never exceeded 15–25% of the maximum level possible [27].

We therefore established a sensitive, TetR-based genetic circuit that is effectively induced by a TIP-tagged protein expressed from the target protein’s native locus. This was achieved with a novel synthetic Tet-regulated system (Buerger *et al.*, manuscript in preparation) introduced into the genome of the well-characterized human pathogen *Salmonella enterica* serovar Typhimurium (*S.* Typhimurium) [29]. TetR is constitutively expressed by the promoter of the chloramphenicol acetyltransferase (*cat*) resistance gene and regulates the expression of a reporter gene which can be induced either by tetracyclines or by fusion of the inducing peptide TIP2 to a carrier protein like Thioredoxin 1 (Trx1) [30], [31]. Sensitive induction of TetR by Trx1-TIP2 was only obtained after mutating the TetR-driving promoter to diminish its strength. The resulting very low steady-state level of TetR did not compromise tight repression in the absence of an inducer, but instead greatly improved the response to the inducer.

## Results

### Peptide-mediated Induction is Not Sensitive Enough if TetR is Expressed by the *Cat* Promoter

**Figure 1:**
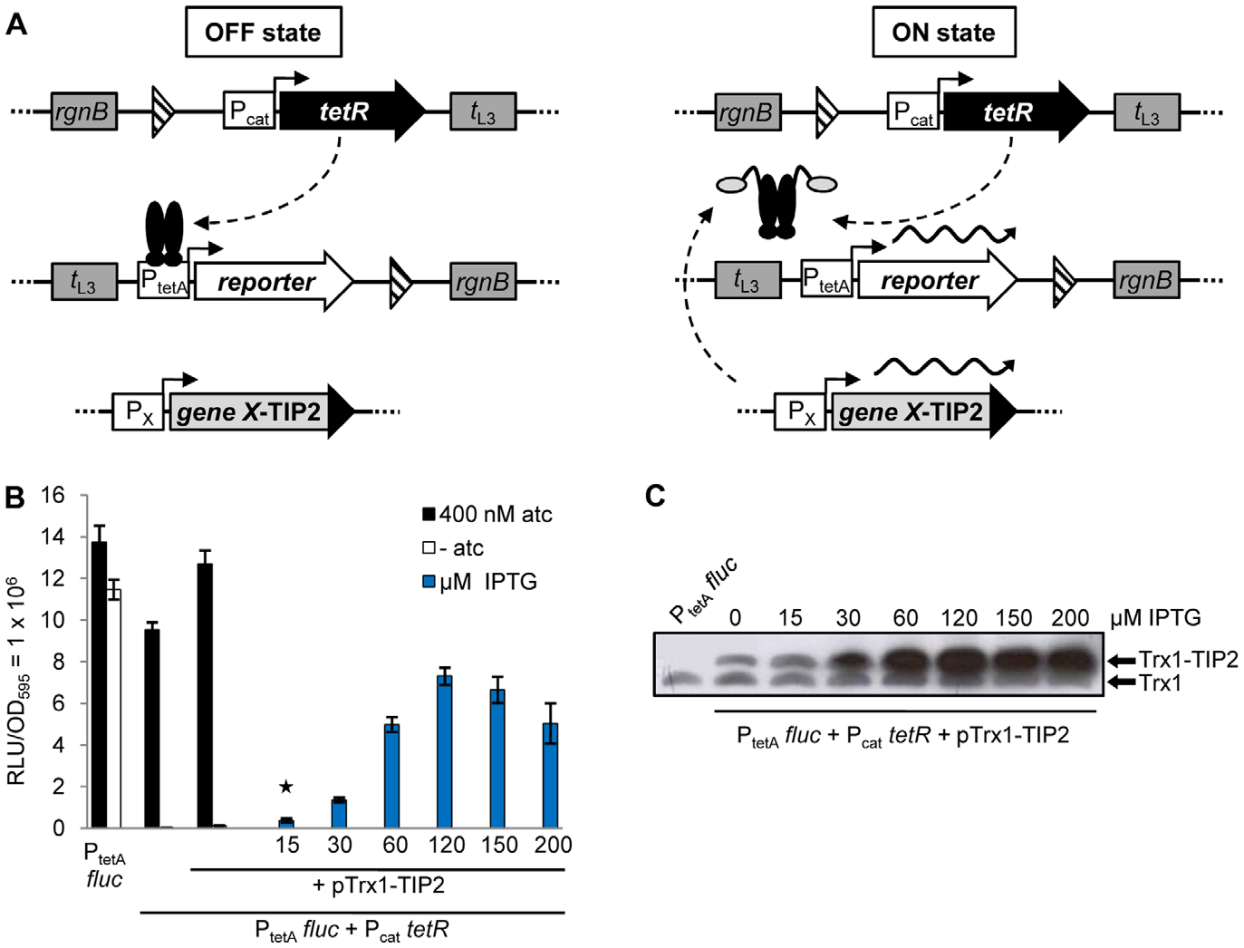
Design of the chromosomally-encoded *Salmonella* reporter system and analysis of its regulatory properties. (A) The *tetR* gene (black arrow) is expressed by P_cat_ (promoters are depicted as white rectangles with arrows on top), the reporter gene (*fluc* or *gfp+*, white arrow) is under control of P_tetA_. They are located in attachment sites of *Salmonella* Genomic Island 1 (2nd *attB*) and phage P22, respectively. TetR (black ovals) is constitutively expressed by P_cat_ and inhibits reporter gene transcription by binding to P_tetA_. If TetR is induced by tetracyclines or, as shown here, by a TIP2 fusion protein (light grey oval with black appendix), the reporter protein is expressed. The translational fusion of TIP2 to a target gene (light grey rectangle with black extension) is expressed either by the *tac* promoter when encoded on a plasmid or by the endogenous promoter when present in the *Salmonella* chromosome. All *Salmonella* reporter strains constructed possess flanking transcription terminators of bacterial (*rgnB*) and phage λ (*t*_L3_) origin (dark grey rectangles) that protect the integrated cassettes against transcriptional read-through. FRT sites are depicted by striped triangles. They remained as scars in the chromosome after excision of the kanamycin resistance cassette following homologous recombination. (B) Luciferase assay of a reporter strain as described in (A) with *fluc* serving as reporter gene, to compare induction of TetR by atc with induction by a plasmid-encoded Trx1-TIP2 fusion protein (pTrx1-TIP2). For maximum induction of TetR, 400 nM atc was added. Trx1-TIP2 expression was induced by increasing the concentration of IPTG. The bars illustrate the relative light units (RLU) which were normalized to a 1 ml culture with OD_595_ = 1. The data are a representative set from at least three independent measurements and display the mean ± standard deviation. (C) Western blot analysis for detection of Trx1-TIP2 steady-state levels with a polyclonal anti-thio antibody. The reporter strain used in (B) was incubated with increasing amounts of IPTG and 5 µg crude protein extract from each sample were loaded onto the gel.

Ideally, a regulatory system for conditional gene expression should not only offer a wide range of expression between its basal and induced states, it should also react sensitively and in a dose-dependent manner to the presence of one or more specific effector(s) [32], [33]. These properties depend, to a great deal, on the components selected to construct the regulatory system and how they are assembled. The Tet-controlled expression system, which was studied here and is displayed schematically in Fig. 1A, is an example of such a synthetic regulatory system. (I) The *cat* promoter (P_cat_) from Tn*9* [34], [35] constitutively expresses the Tet repressor protein (TetR). (II) The reporter gene mRNA, which is transcribed by the Tet-regulated promoter of the resistance gene *tetA* (P_tetA_), contains a modified 5′ untranslated region. (III) Each expression cassette was inserted in single copy at a different attachment site in the *Salmonella* genome. (IV) TetR is induced either by tetracycline derivatives, or by an artificially selected TetR-inducing peptide (TIP), fused to a plasmid- or chromosomally-encoded carrier protein.

Because the regulatory properties of the strain carrying this new and artificial genetic circuit were unknown, we analyzed the inducibility of TetR using both types of effector − anhydrotetracycline (atc) representing a potent natural inducer [36], and the peptide TIP2 [21] fused to the C-terminus of Thioredoxin 1 (Trx1, *trxA*) as alternative inducer representing a signal transduction pathway. The Trx1-TIP2 fusion is expressed by the *tac* promoter and, thus, under transcriptional control of Lac repressor [37]. This expression cassette is encoded on a plasmid which was introduced into the strain containing P_tetA_ *fluc* and P_cat_ *tetR*. The strain was incubated with atc or with increasing IPTG concentrations to induce the Lac repressor and, concomitantly, expression of Trx1-TIP2 to see if TIP2 is as active as atc in inducing TetR. Fig. 1B shows that this was not the case. While atc fully induced luciferase expression to the level observed for the control strain lacking TetR (P_tetA_ *fluc*, first set of bars), Trx1-TIP2 expression, in contrast, did not lead to luciferase activity exceeding 50% of the maximum level. In addition, 120 µM IPTG were needed to reach this level, a concentration that fully induces the *tac* promoter [19], [37]–[39]. Although the RLU did increase with rising IPTG concentrations, the luciferase activity observed at lower amounts of IPTG (15, 30 µM) was only very weak.

We then analyzed the expression of Trx1-TIP2 in a Western blot. It confirmed that higher IPTG concentrations led to higher steady-state levels of the fusion protein (Fig. 1C). Maximum levels were observed for cultures grown with 120 µM IPTG or more. In the absence of IPTG, expression of the fusion protein was also detected, most likely due to leakiness of the *tac* promoter transcribing *trxA-TIP2* [20], [37]. This would explain the 5.4-fold higher basal luciferase activity detected in strains transformed with the plasmid (Fig. 1B, second and third set of bars). The amount of Trx1-TIP2 expressed after adding 15 µM IPTG (lane 3, Fig. 1C) is roughly identical to the endogenous steady-state level of Trx1 (lower band, Fig. 1C). At this concentration, however, there was no noticeable induction of TetR (marked with a star in Fig. 1B).

Combined with the observation that Trx1-TIP2 did not fully induce TetR, despite maximum expression by a strong promoter, we concluded that peptide-mediated induction of TetR is not sufficient in this strain if the tagged proteins are expressed at low or intermediate levels. For this to happen, would require dramatic improvement in the dose-response curve, but without compromising tight repression of reporter gene transcription. An important aspect that contributes to the sensitivity of induction is the expression level of the repressor itself [19], [32], [40]–[42]. High levels of a repressor require higher concentrations of the inducer and can even interfere with induction. Hence, the *cat* promoter driving TetR expression was mutated to make it less active.

### Directed Mutagenesis of the P_cat_ -10 Element Leads to Improved Sensitivity of TIP2-mediated TetR Induction

**Figure 2:**
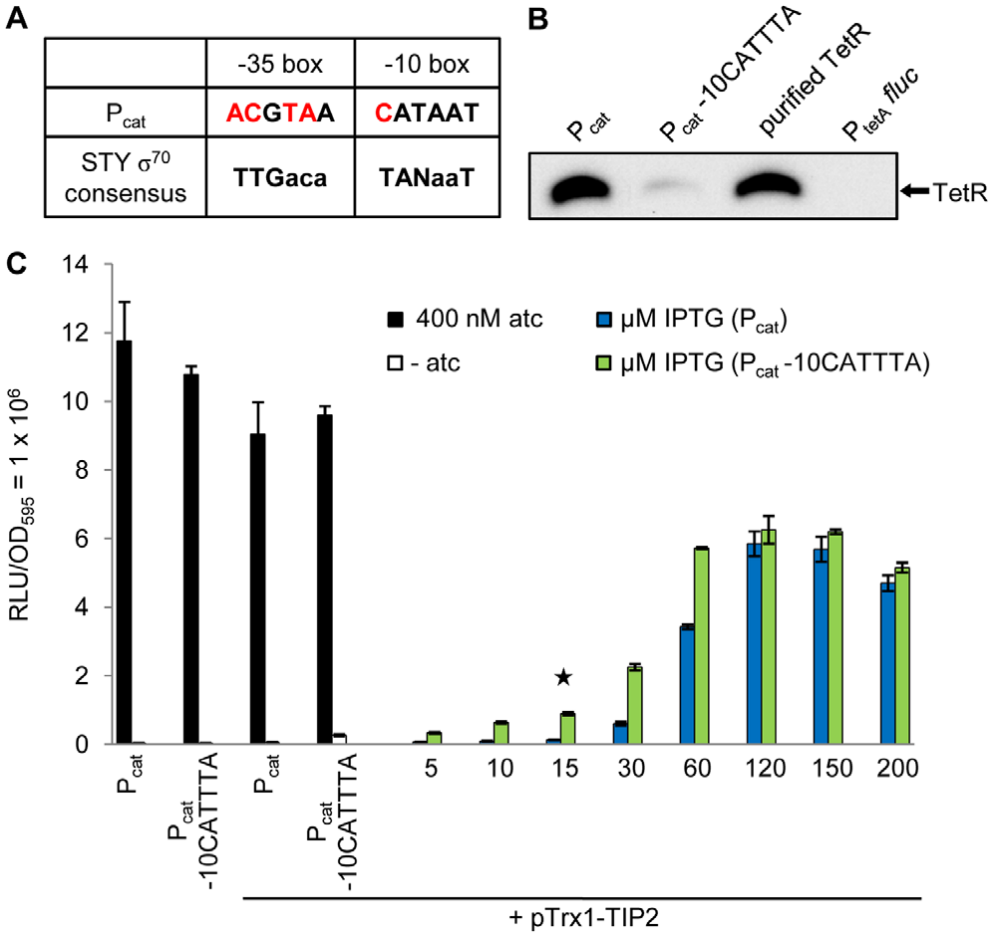
Analysis of the reporter system’s regulatory properties after directed mutagenesis of the P_cat_ -10 element. (A) Comparison of the P_cat_ and the *S.* Typhimurium (STY) σ^70^ consensus promoter sequences. Differences are highlighted in red. (B) Western blot for determining the steady-state level of TetR expressed by P_cat_ or P_cat_ -10CATTTA (5 µg crude protein extract of each) with a polyclonal anti-TetR antibody. As controls, 5 µg crude protein extract of the strain P_tetA_ *fluc* (lacking TetR) and 30 ng of purified TetR were loaded onto the gel. (C) Dose-response curve to analyze the sensitivity of TetR induction by the plasmid-encoded Trx1-TIP2 fusion protein (pTrx1-TIP2) in strains expressing TetR either by P_cat_ or by P_cat_ -10CATTTA. These were incubated without and with 400 nM atc as control for maximum induction of TetR. Increasing IPTG concentrations were added for Trx1-TIP2 expression. The star marks the IPTG concentration at which the level of Trx1-TIP2 corresponds roughly to the endogenous Trx1 level. The bars illustrate the relative light units (RLU) which were normalized to a 1 ml culture with OD_595_ = 1. The data are a representative set from at least three independent measurements and display the mean ± standard deviation.

As shown in Fig. 2A, the annotated -10 element of the Tn*9 cat* promoter [35], [43], [44] has 83%, the -35 element only 33% sequence identity to the *S.* Typhimurium σ^70^ consensus promoter sequence [45], which closely matches the -35 and -10 elements of the *E. coli* σ^70^ consensus sequence [46]–[48]. Mutations reducing the identity of these elements to the consensus sequence negatively affect promoter activity [46], [47], [49]. Since the identity of the -35 element to the consensus sequence is already quite poor, we mutated only the -10 element by inverting the last three nucleotides (positions -8, -9 and -10) from “CAT**AAT**” to “CAT**TTA**”. This destroys the consensus sequence at these positions, but retains the element’s GC content. The mutation was introduced by homologous recombination into the chromosome of the strain WH1102 containing P_tetA_ *fluc* and P_cat_ *tetR*.

To analyze if the mutation had affected the steady-state level of TetR, Western blots were performed (Fig. 2B). The strain without TetR (P_tetA_ *fluc*) was again used as control. The signal for TetR was indeed strongly reduced in the strain with the promoter mutation (Fig. 2B, lanes 2 and 1, respectively). We then determined if this reduction resulted in increased sensitivity towards the peptidic inducer. The strains expressing TetR either by wildtype P_cat_ or by the weaker, mutated P_cat_ -10CATTTA were transformed with the plasmid encoding the Trx1-TIP2 fusion and their luciferase activities assayed (Fig. 2C). Despite the strong reduction in the TetR steady-state level, repression by TetR was not affected, since the luciferase activities were similar in both strains in the absence of inducer (Fig. 2C, first set of bars: 34314±904 RLU and 35464±1791 RLU, respectively). However, we detected higher luciferase activity in the mutant strain at low and intermediate concentrations of IPTG (from 5 to 60 µM, with 7.3-fold higher luciferase activity at 15 µM, highlighted by a star in Fig. 2C) compared to the strain with the wildtype promoter. Although the sensitivity of peptide-mediated induction was clearly improved, the maximum possible expression level of the reporter system, indicated by atc-mediated induction of TetR, was not reached by TIP2-induced reporter gene expression in the P_cat_ -10CATTTA strain. More importantly, levels of Trx1-TIP2 corresponding to the endogenous Trx1 level still did not lead to efficient induction of TetR. In this context, a genetic network relying on chromosomal expression of Trx1 as signal for induction of TetR would not be functional.

### Random Mutagenesis of the P_cat_ -10 Element Yields Promoter Mutants with Sensitive TetR Induction

**Figure 3:**
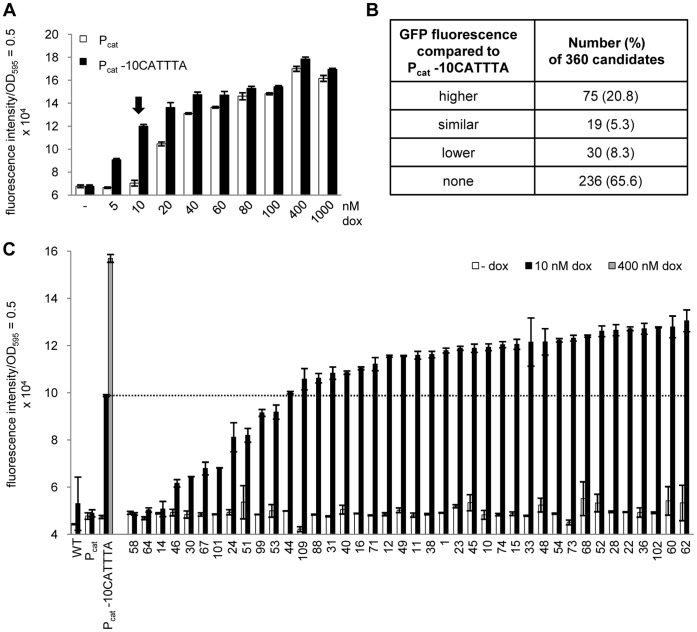
A synthetic promoter library generated by random mutagenesis of the P_cat_ -10 element yields candidates with different induction efficiency. (A) Dose-response curve of P_cat_ and P_cat_ -10CATTTA strains to identify the dox concentration for screening (indicated by an arrow). Both strains were incubated with increasing amounts of dox, and their respective GFP fluorescence was determined in a microplate reader. (B) Classification of the 360 library candidates according to their GFP fluorescence with respect to the reference strain carrying P_cat_ -10CATTTA. Total numbers are listed and percentages are given in brackets. (C) GFP fluorescence measurement of 40 promoter library candidates, ordered by increasing fluorescence. Controls were *Salmonella* WT and the strains containing P_tetA_ *gfp+* either with P_cat_ *tetR* or with P_cat_ -10CATTTA *tetR*. Candidates and controls were incubated without and with 10 nM dox. For maximum induction of TetR, the P_cat_ -10CATTTA strain was incubated with 400 nM dox. The dashed line allows a direct comparison with the induction level of the P_cat_ -10CATTTA strain at 10 nM dox. Bars in (A) and (C) represent the fluorescence intensity which was normalized to a 1 ml culture with OD_595_ = 0.5. The data are a representative set from at least three independent measurements and display the mean ± standard deviation.

To further improve the system’s regulatory properties, we generated a library of promoter variants. Such a pool should offer a broad range of promoter activities and, thus, different TetR induction levels and sensitivities [50], [51]. First, the reporter system was adapted to allow fast and easy screening of induction in living cells by exchanging the *fluc* reporter gene against *gfp+* [52]. The resulting strains WH1104 and WH1106 carrying P_tetA_ *gfp+* and P_cat_ *tetR* or P_cat_ -10CATTTA *tetR*, respectively, served as controls in the promoter library screens. Because atc is very light-sensitive, we switched to doxycycline (dox) for screening. The ideal dox concentration should not lead to induction of TetR in the strain with P_cat_, but should result in intermediate activity in the P_cat_ -10CATTTA mutant to allow sensitive detection of differences in inducibility. After incubating both strains with increasing amounts of dox (Fig. 3A), we observed maximum induction at 400 nM dox, in agreement with data from *fluc* reporter strains. At 10 nM dox (indicated by an arrow in Fig. 3A), the P_cat_ strain still had background GFP fluorescence, while the activity detected in the P_cat_ -10CATTTA strain was 67% of the maximum fluorescence measured. This dox concentration was therefore used for screening.

The promoter library was generated by random substitution of the last four nucleotides in the P_cat_ -10 element (CA**NNNN**, positions -8 to -11). It was introduced into the strain WH1104 containing P_tetA_ *gfp+* and P_cat_ *tetR*, with mutant fragments replacing P_cat_ at the chromosomal level by homologous recombination [53]. A total number of 360 candidates were assayed for GFP fluorescence to analyze their TetR inducibility. They, as well as the controls − *Salmonella* WT and the strains containing P_tetA_ *gfp+* either with P_cat_ *tetR* or P_cat_ -10CATTTA *tetR* − were incubated without and with 10 nM dox. The P_cat_ -10CATTTA strain was also incubated with 400 nM dox to define the maximum level of GFP fluorescence. Approximately two-thirds of the candidates displayed no increase in reporter activity after incubation with 10 nM dox. The other third showed very different GFP fluorescence intensities and was grouped roughly in Fig. 3B. The data obtained for 40 clones serves as an example for the diverse reporter activities of the candidates (Fig. 3C). The dashed line highlights candidates with improved TetR induction compared to the P_cat_ -10CATTTA strain. Most importantly, all candidates still fully repressed reporter gene transcription.

**Figure 4:**
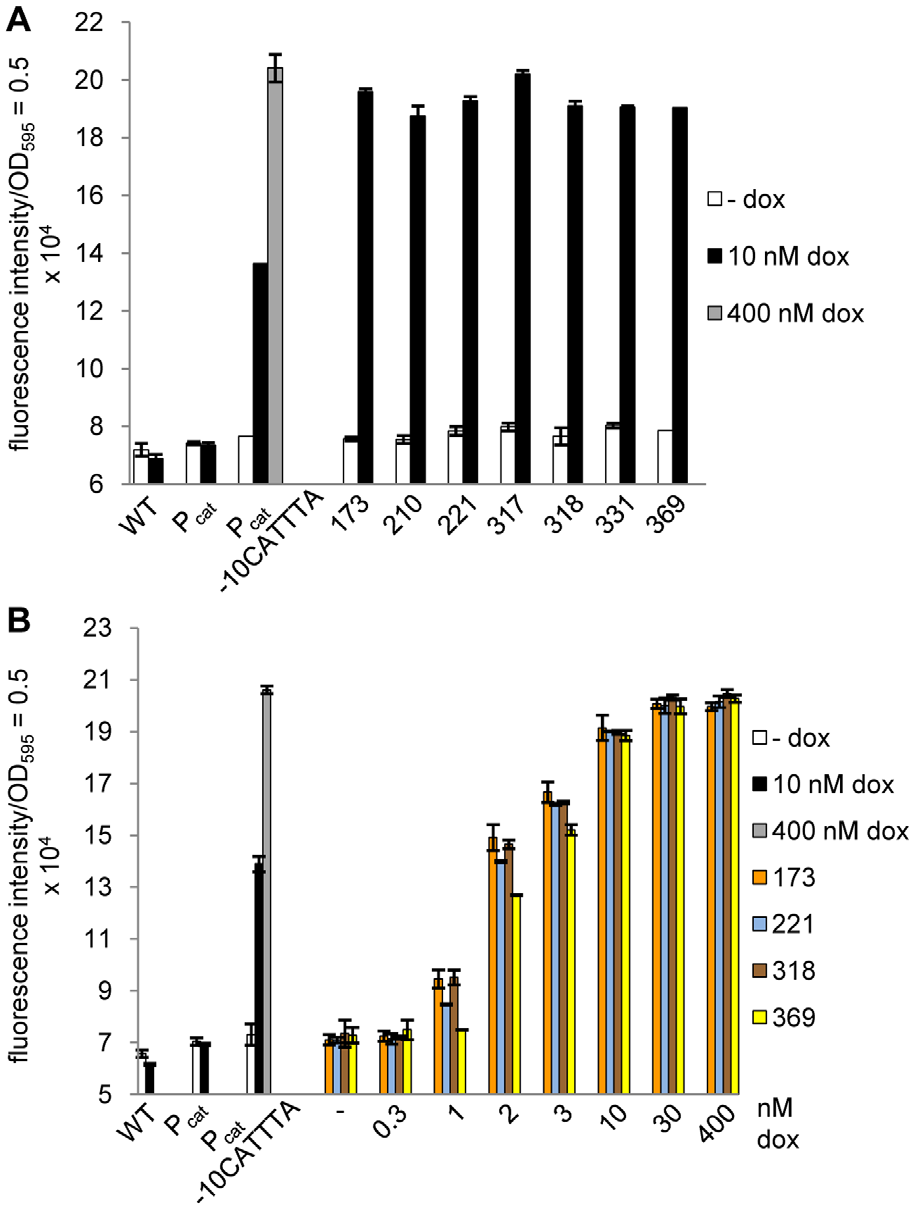
Identification of promoter mutants with increased TetR inducibility. (A) GFP fluorescence measurement of the seven promoter mutants with the highest reporter activity. (B) Dose-response curve of four promoter variants possessing different -10 elements to analyze the sensitivity of TetR induction at increasing dox concentrations. For both data sets, controls and cultivation were the same as in Fig. 3C. Bars illustrate the fluorescence intensity which was normalized to a 1 ml culture with OD_595_ = 0.5. The data are a representative set from at least three independent measurements and display the mean ± standard deviation.

The number of candidates was step-wise reduced to the set of seven shown in Fig. 4A which displayed the best regulatory properties. With only 10 nM dox, they almost reached the fluorescence level the P_cat_ -10CATTTA mutant needed a 40-fold higher dox concentration for. Sequence analysis of their -10 elements revealed that all were GC-enriched, carrying either four (#318), three (#173, #317, #331, #369) or two (#210, #221) G/C nucleotides at the four randomly mutated positions (Table S1 in Text S1). The -10 elements of the candidates #173, #317 and #331 were identical (CAGCCA). For these three independently isolated promoter mutants, we observed a similar response to increasing dox concentrations (Fig. S1), indicating that the induction determined was a property of the promoter mutation and not of unknown mutations in the genome. A dox titration was performed with candidate #173 and three other candidates carrying different -10 elements to identify the one with the best TetR inducibility at low effector concentrations (Fig. 4B). Candidates #173 and #318 showed slightly higher GFP fluorescence at 1 to 3 nM dox. At 30 nM dox, all promoter mutants had already reached the level of maximal reporter gene expression, which requires a 13-fold higher dox concentration in the P_cat_ -10CATTTA strain. Taken together, the synthetic promoter library yielded several variants with improved sensitivity towards dox. For the following experiments, candidate #173 (P_cat_ -10CAGCCA) was selected, because it revealed slightly higher reporter activity compared not only to the other two candidates carrying the same -10 element (Fig. S1), but also to the candidates with the different -10 elements (Fig. 4B).

### The Sensitivity of TetR Induction in the P_cat_ Promoter Variants Correlates with the Steady-state Level of TetR

**Figure 5:**
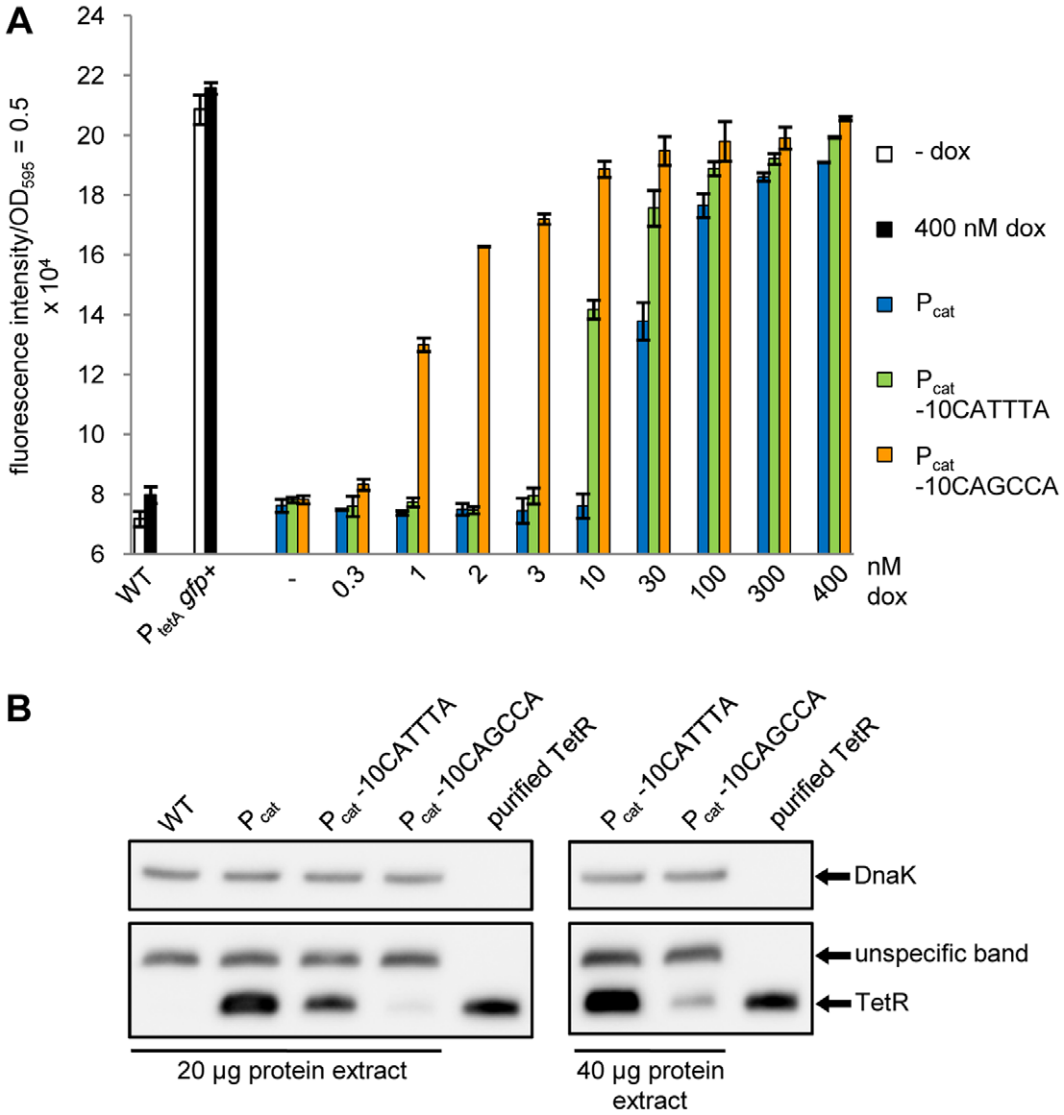
Comparison of the strains with the promoters P_cat_, P_cat_ -10CATTTA or P_cat_ -10CAGCCA expressing TetR. (A) Dose-response curve of the promoter variants (P_tetA_ *gfp+* with P_cat_/P_cat_ -10CATTTA/P_cat_ -10CAGCCA *tetR*) which were incubated with increasing dox concentrations. The control strains, *Salmonella* WT and the strain lacking TetR resulting in constitutive GFP expression (P_tetA_ *gfp+*), were incubated without inducer or with 400 nM dox for maximum reporter activity. The bars illustrate the fluorescence intensity which was normalized to a 1 ml culture with OD_595_ = 0.5. The data are a representative set from at least three independent measurements and display the mean ± standard deviation. (B) Western blot analysis of the steady-state levels of TetR expressed either by P_cat_, P_cat_ -10CATTTA or P_cat_ -10CAGCCA, detected with a polyclonal anti-TetR antibody. *Salmonella* WT and 20 ng of purified TetR served as controls. For each strain, 20 µg crude protein extracts were loaded (left panel). Additionally, 40 µg crude protein extract from the mutants P_cat_ -10CATTTA and P_cat_ -10CAGCCA were also analyzed (right panel). DnaK served as loading control in both blots and was detected with a monoclonal anti-DnaK antibody.

We compared the sensitivity of TetR induction in the strains with the promoter variants P_cat_, P_cat_ -10CATTTA or P_cat_ -10CAGCCA by incubating them with increasing concentrations of dox (Fig. 5A). As additional control, a strain lacking TetR (WH1136 which carries only P_tetA_ *gfp+*) was generated to define the maximum GFP fluorescence intensity possible. With only 1 nM dox, the P_cat_ -10CAGCCA strain displayed nearly half-maximal GFP fluorescence, which required incubation with a 10-fold higher dox concentration for the strain carrying P_cat_ -10CATTTA or a 30-fold higher concentration for the strain with P_cat_. Even at the maximum dox concentration, the P_cat_ -10CAGCCA strain showed slightly higher GFP fluorescence than the other promoter variants and almost reached the level of the reporter strain lacking TetR. Taken together, we demonstrated step-wise improvement in the dose-response of TetR induction towards lower dox concentrations from P_cat_ via P_cat_ -10CATTTA to P_cat_ -10CAGCCA.

We then examined if the increased sensitivity of TetR induction in the P_cat_ -10CAGCCA strain correlated with a further reduction in the intracellular amount of TetR (Fig. 5B). As for the corresponding *fluc* expressing reporter strain, the P_cat_ -10CATTTA *gfp* reporter strain showed a reduced steady-state level of TetR compared to the strain with P_cat_ (Fig. 5B, lanes 2 and 3). For the strain in which TetR transcription is mediated by P_cat_ -10CAGCCA, we observed an even weaker signal for TetR compared to the other strains. The TetR-specific band was barely visible when the same amounts of crude protein extract were loaded for all promoter variants. A proper TetR signal in the P_cat_ -10CAGCCA strain was only seen when the amount of protein extract was doubled. We concluded that the mutation strongly affected promoter activity resulting in a massive reduction of intracellular TetR. Surprisingly, this mainly improved the sensitivity of induction, but rather did not compromise efficient repression of reporter gene transcription by TetR. To confirm this, we measured reporter gene expression in a more sensitive approach using a spectrofluorometer (Fig. S2). Maximum induction determined with the plate reader was about 2.5-fold. In the spectrofluorometer, it increased to approximately 25-fold (Fig. S2). Still, background green fluorescence in the repressed state changed only from 23373±215 fluorescence units (100%) with wildtype P_cat_ to 23971±651 fluorescence units (103%) with the weaker promoter P_cat_ -10CATTTA and 26322±274 fluorescence units (113%) with the weakest promoter, P_cat_ -10CAGCCA. If at all, repression mediated in the strain containing this promoter is negligibly weaker than in a strain with the wildtype promoter.

Additionally, we performed growth curves in LB as a rich medium and in LPM, pH5.8, as a minimal medium (Fig. S3), confirming that neither the introduction of the genetic elements into the genome, nor the constitutive expression of TetR had affected growth of the reporter strains compared to WT *Salmonella*.

### A Chromosomally Encoded Trx1-TIP2 Fusion Protein Induces TetR Efficiently only in the Most Sensitive Strain with P_cat_ -10CAGCCA

After comparing TetR induction by dox in the promoter variants P_cat_, P_cat_ -10CATTTA and P_cat_ -10CAGCCA, we examined if the sensitivity of peptide-mediated induction by TIP2 was also improved in the P_cat_ -10CAGCCA mutant.

**Figure 6:**
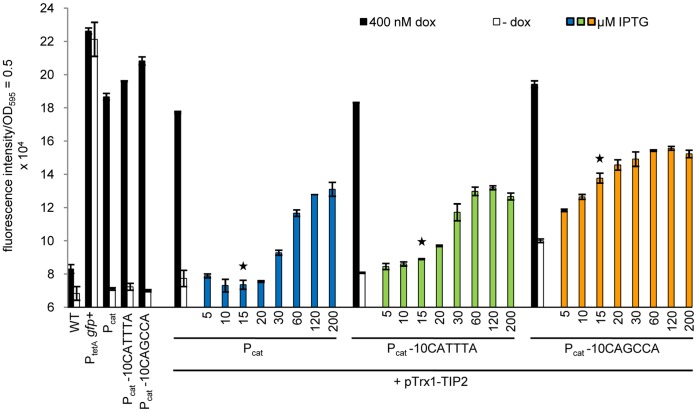
Dose-response curve of TetR induction by a plasmid-borne Trx1-TIP2 fusion protein in the strains with promoters P_cat_, P_cat_ -10CATTTA or P_cat_ -10CAGCCA expressing TetR. The promoter variants were incubated without and with 400 nM dox for maximum induction of TetR or with increasing IPTG concentrations for plasmid-encoded Trx1-TIP2 (pTrx1-TIP2) expression. The *Salmonella* WT strain, the reporter strain lacking TetR (P_tetA_ *gfp+*) and the promoter mutants without the plasmid served as controls and were incubated without and with 400 nM dox. The star marks the IPTG concentration at which the level of Trx1-TIP2 corresponds roughly to the endogenous Trx1 level. The bars represent the fluorescence intensity which was normalized to a 1 ml culture with OD_595_ = 0.5. The data are a representative set from at least three independent measurements and display the mean ± standard deviation.

First, the three promoter variants were transformed with the plasmid carrying the IPTG-inducible Trx1-TIP2 expression construct. Cultures of the transformed strains were incubated with dox or IPTG and their GFP fluorescence analyzed (Fig. 6). For the strains with P_cat_ or P_cat_ -10CATTTA driving TetR expression, increased fluorescence was not observed until incubation with 30 or 20 µM IPTG, respectively. The P_cat_ -10CAGCCA strain reacted much more sensitively towards the presence of Trx1-TIP2, because it displayed elevated GFP fluorescence already without adding IPTG. Hence, we only saw efficient induction of TetR at Trx1-TIP2 levels corresponding to the endogenous Trx1 level in the P_cat_ -10CAGCCA mutant: at 15 µM IPTG (highlighted by a star in Fig. 6), this strain revealed an increase in signal strength of about 97% compared to its fluorescence in the absence of any inducer (Fig. 6, first set of bars), whereas the increase for the strain with P_cat_ was only about 4% or 23% for the strain with P_cat_ -10CATTTA.

**Figure 7:**
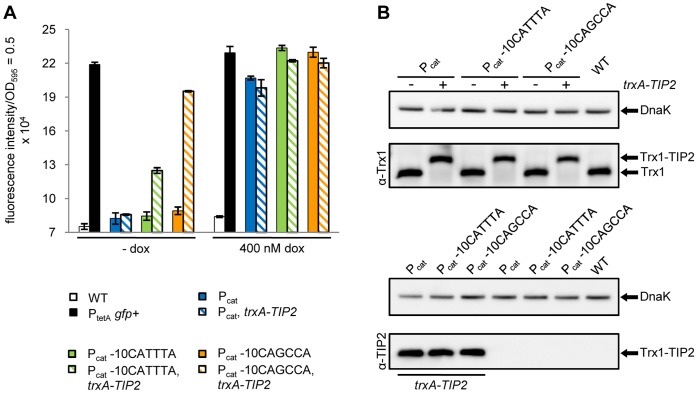
TetR induction by endogenous levels of a Trx1-TIP2 fusion in the promoter variants P_cat_, P_cat_ -10CATTTA and P_cat_ -10CAGCCA. (A) GFP fluorescence measurement to examine TetR induction by a chromosomally encoded Trx1-TIP2 fusion protein. The three promoter variants and the control strains − *Salmonella* WT, the reporter strain lacking TetR (P_tetA_ *gfp+*) and the promoter variants without TIP2 in the genome − were incubated without and with 400 nM dox. The bars illustrate the fluorescence intensity which was normalized to a 1 ml culture with OD_595_ = 0.5. The data are a representative set from at least three independent measurements and display the mean ± standard deviation. (B) Western blot for determining the steady-state levels of endogenous or TIP2-tagged Trx1 in the three promoter variants. The proteins were detected by polyclonal antibodies against either Trx1 (top) or TIP2 (bottom). With *Salmonella* WT serving as control, 10 µg crude lysate of each strain were loaded onto the gels. DnaK served as loading control and was detected with a monoclonal anti-DnaK antibody.

This prompted us to chromosomally tag endogenous *trxA* with TIP2 by fusing its coding sequence to the 3′ end of the *trxA* gene in the genomes of all three promoter variants. TetR induction by the fusion protein was determined by measuring GFP fluorescence and compared between the promoter variants (Fig. 7A). As control, we also incubated both TIP2-tagged and non-tagged parental strains with 400 nM dox to see if Trx1-TIP2-mediated induction was as effective as dox-mediated induction. For P_cat_ -10CAGCCA, this was nearly the case (fourth set of bars in Fig. 7A), because its GFP fluorescence reached about 85% of both the maximal GFP fluorescence possible as well as the maximum dox-induced level. In contrast, the TIP2-tagged P_cat_ -10CATTTA mutant did not even reach the half-maximum level of dox-mediated TetR induction. The P_cat_ strain did respond to dox, but revealed no induction of TetR by chromosomally encoded Trx1-TIP2 at all. This agrees with our observation that only the P_cat_ -10CAGCCA mutant allowed efficient induction by plasmid-encoded Trx1-TIP2 when the fusion protein was expressed at a level similar to the endogenous level of Trx1. Overall, these data indicate that manipulating the sequence of the promoter expressing TetR greatly affected its sensitivity of induction not just for a natural inducer, but also for an artificial peptidic inducer.

Because we wanted to ascertain that the TIP2-tag did not interfere with the Trx1 steady-state level, we determined the endogenous Trx1 level in the untagged strains, as well as the Trx1-TIP2 fusion protein levels in the respective TIP2-tagged strains for all three promoter variant strains. The corresponding Western blots are depicted in Fig. 7B. The Trx1 steady-state levels were not altered in the promoter variants and similar to the level in the *Salmonella* WT. The steady-state levels of the TIP2-tagged proteins appeared slightly reduced with decreasing promoter strength in the strains carrying P_cat_, P_cat_ -10CATTTA and P_cat_ -10CAGCCA, respectively, although this was not observed consistently in all blots. Besides, bands detected with the TIP2-specific antibody did not reveal any differences between these three strains. But, even if the Trx1-TIP2 levels were slightly reduced, induction in the promoter mutants was still higher than in the strain carrying the wildtype promoter expressing TetR. This further emphasizes that low-level expression of a regulatory protein is key to the functionality of this peptide-induced genetic circuit.

## Discussion

A hallmark of inducible gene expression systems is their dose-dependent response to one or more specific effectors. In bacteria, these are frequently small molecules. Roughly 70% of the known regulatory interactions in *E. coli*, for example, are modulated by transcription factors directly sensing signal metabolites [54] and many of these have been intensely studied [3], [55]–[57]. Although less common and less-well characterized, gene expression is also controlled in bacteria by the protein-protein interactions that take place in signal transduction cascades [24]–[26], [58], [59]. So far, most applications that utilize inducible gene expression have relied on transcription factors controlled by small molecules, like AraC, LacI or TetR [15], [16]. The recent isolation of peptide-based inducers of TetR [19]–[21] now allows us to address the question if and how a model repressor can also function as a regulator of gene expression in an artificial genetic circuit, if it is regulated by a protein carrying an inducing peptide and is, thus, part of a signal transduction pathway.

We studied this by stably integrating an artificial regulatory circuit in single copy into the genome of the human pathogen *S.* Typhimurium. In principle, such a synthetic biology approach allows to generate any kind of regulatory system just by combining a set of components from prior existing building blocks, taken either from natural systems [60] or artificially synthesized [61]. Frequently, these artificial systems still require some degree of refinement before they achieve the desired activity. This was also the case here, because the regulatory system initially did not respond sensitively to its peptidic inducer (Fig. 1). We assumed this to be due to high-level expression of TetR by the promoter and the 5′ untranslated region of the *cat* resistance gene and therefore reduced the promoter’s activity, particularly by modifying its -10 element. Alternatively, we could have tried to modulate TetR expression by modifying its ribosome binding site (RBS). However, we decided against this approach, because controlling the efficiency of translation is not just a simple function of the RBS sequence, but also depends on the distance between RBS and start codon and on the mRNA secondary structure [62]. Because a directed promoter mutagenesis (P_cat_ -10CATTTA) improved system inducibility only marginally, we also abandoned this approach, because it would have resulted in tedious trial-and-error testing of an unknown number of base-specific mutations, and instead screened a synthetic promoter library. Libraries of artificial promoters have been generated in different microorganisms including *E. coli* [51], [63], [64], lactic acid bacteria [50], [65] and yeasts [66]–[68] and cover a wide range of promoter strengths in small steps. But they were mostly established and characterized at the plasmid level. Here, we created a chromosomally located promoter library, because this more closely represents the physiological situation of natural regulatory circuits. This unbiased approach also yielded a broad range of promoter activities, as the 40 different promoter mutants shown in Fig. 3C clearly demonstrate.

The promoter library candidates were analyzed and grouped with respect to their sensitivity of TetR induction. The best seven candidates all had GC-enriched -10 elements containing up to five G/C basepairs. P_cat_ -10CAGCCA, the most sensitive promoter variant, carries 4 G/C basepairs in its -10 box. In contrast, the -10 hexamers of *E. coli* promoters analyzed by Harley and Reynolds [48] did not have more than three G/C basepairs and even these represented a mere 10 out of the 263 promoters surveyed. Feklistov and Darst [69] structurally elucidated the decisive role of an “A” nucleotide at position -11 in promoter recognition and strand separation. The importance of this nucleotide had previously been described in several other studies [70]–[73]. This nucleotide was retained in our promoter mutants and might be one reason why the promoter was active at all, despite the complete loss of all other sequence identity. Their structural analysis additionally emphasized that promoter recognition by the σ subunit of the RNA polymerase holoenzyme and DNA melting are closely coupled in the same process and highly sequence-dependent [69]. Thus, a reduced identity of the -10 element to the consensus sequence “TATAAT” negatively affects promoter activity, most likely by interfering with this coupled binding and unwinding process. This has been shown by both sequence [46]–[48] and genetic analysis [50], [74], [75], by chemical and enzymatic probing [49] and by oligonucleotide binding to the RNA polymerase holoenzyme [71], [76], [77]. Taken together, we infer that the shift from an AT- to a GC-rich -10 element in our promoter mutants negatively affects promoter recognition and DNA melting by i) destroying the near-consensus sequence and, hence, eliminating important contact sites to RNA polymerase, and ii) stabilization of the DNA duplex by the increased number of hydrogen bonds. The consequence is a strongly reduced steady-state level of TetR which in turn enables its sensitive induction. The higher degree of degeneracy observed for the σ^70^ promoter consensus sequence in *S.* Typhimurium as opposed to *E. coli* [45] might be a second reason why we see weak promoter activity for the GC-rich -10 elements from our library.

That the regulatory properties of repressed systems can depend to a large extent on the repressor’s intracellular level has been observed before. Bertrand and colleagues found that the concentration of tetracycline required for induction of TetR directly correlated with the TetR expression level [42]. The steady-state level of TetR also affected the sensitivity of TIP-mediated induction [19]. Even in a synthetic Tet-regulated expression system in the yeast *Saccharomyces cerevisiae* with its different mechanism of controlling gene expression, reduced levels of TetR, obtained by introducing specific mutations in the TATA box of the TetR driving promoter, led to an increase in the sensitivity of induction [78]. This general correlation is not limited to TetR, but has also been found for other repressor proteins, like LacI [79] or the lambda cI857 repressor [80]. At least as important as the increased sensitivity of induction was the observation that the dramatically reduced intracellular amount of TetR did not affect the repression of reporter gene transcription at all. This might be due to TetR being an extremely efficient repressor resulting from the high specificity of TetR for *tetO* over non-specific DNA [8]. As consequence, TetR tightly represses gene transcription even if present in very low concentrations in the cell, thus, providing a stable OFF state in the absence of inducer. Repression by TetR has to be tight in the natural context of tetracycline resistance, because overproduction or constitutive expression of the resistance protein TetA strongly reduces bacterial fitness [5], [6]. However, for tetracycline resistance to function, sensitive induction of TetA expression is also crucial for the cell to ensure that the resistance protein is translated before the antibiotic reaches an intracellular level that inhibits translation [7]. By mutating P_cat_, we achieved this sensitive response not just towards low concentrations of natural TetR effectors. More important is the observation that the most sensitive TetR-controlled strain (P_cat_ -10CAGCCA) is also efficiently induced by the alternative effector − a fusion protein of the constitutively expressed housekeeping protein Trx1 [81], [82] with the TetR-inducing peptide TIP2, either expressed from a plasmid (Fig. 6) or from its genomic locus at its endogenous level (Fig. 7A). Thus, we show that a signal transduction cascade can induce TetR-controlled gene expression, but that the regulatory system must react very sensitively to the presence of the peptidic inducer.

In conclusion, the sensitivity of the regulatory circuit assembled in this work is primarily determined by the strength of the promoter driving expression of TetR. Thus, by manipulating the intracellular level of TetR solely through promoter mutation, we not only achieved the improved sensitivity of our novel reporter system that allowed us to establish a regulatory circuit which is triggered effectively by the expression of an endogenous protein. This regulatory system can now be used as a model to set up signal transduction networks for peptide-mediated regulation of gene expression and thereby simulate biological signaling. This is gaining increased attention considering the many approaches used to obtain novel peptides that bind and regulate a target protein’s activity [83]–[87]. Moreover, the P_cat_ -10CATTTA and P_cat_ -10CAGCCA mutants, as well as other promoter variants from our library, can be used in different genetic networks to fine-tune the expression of a respective target gene, thereby adding a new instrument to the genetic and synthetic engineering toolbox.

## Materials and Methods

### Bacterial Strains, Plasmids and Culture Conditions

The strains and plasmids used in this study are listed in Tables 1 and 2, respectively. *Salmonella enterica* serovar Typhimurium strain NCTC 12023 (ATCC 14028s) served as wildtype strain (WT), and strain LB5000 as shuttle strain for plasmids isolated from *Escherichia coli*. Bacterial strains were routinely grown in liquid broth (LB) after Miller [88] at 37°C and 190 rpm or on LB agar plates containing antibiotics if required. Antibiotics were added to the following final concentrations: 100 µg/ml ampicillin, 100 µg/ml kanamycin for *S.* Typhimurium or 60 µg/ml kanamycin for *E. coli*, 25 µg/ml chloramphenicol.

**Table 1:** Bacterial strains used in this study.

Strain	Relevant characteristic(s)	Source or reference
*E. coli* K-12 BW25141	*lacI* ^q^, *rgnB*_T14_, Δ*lacZ*_WJ16_, Δ*phoBR*580, *hsd*R514, Δ*araBAD*_AH33_, Δ*rhaBAD*_LD78_, *galU*95, *endA*_BT333_,*uidA*(Δ*mlu*I)::*pir* ^+^, *recA*1	[53]
*S.* Typhimurium NCTC 12023	WT	
*S.* Typhimurium LB5000	r_LT_^−^ m_LT_^+^, r_SA_^−^ m_SA_^+^, r_SB_^−^ m_SB_^+^, *metA*22, *metE*551, *trpD*2, *leu*	[96]
WH1001	NCTC 12023; P22*attB* a::*rgnB*, *FRT*, P_tetA_ *fluc*, *FRT*, *t*_L3_	This study
WH1102	NCTC 12023; P22*attB*::*rgnB*, *FRT*, P_tetA_ *fluc*, *FRT*, *t*_L3_; SGI1*attB* b::*rgnB*, *FRT*, P_cat_ *tetR*, *t*_L3_	This study
WH1109	NCTC 12023; P22*attB*::*rgnB*, *FRT*, P_tetA_ *fluc*, *FRT*, *t*_L3_; SGI1*attB*::*rgnB*, *FRT*, P_cat_ -10CATTTA *tetR*, *t*_L3_	This study
WH1104	NCTC 12023; P22*attB*::*t*_L3_, P_tetA_ *gfp+*, *FRT*, *rgnB*; SGI1*attB*::*rgnB*, *FRT*, P_cat_ *tetR*, *t*_L3_	This study
WH1106	NCTC 12023; P22*attB*::*t*_L3_, P_tetA_ *gfp+*, *FRT*, *rgnB*; SGI1*attB*::*rgnB*, *FRT*, P_cat_ -10CATTTA *tetR*, *t*_L3_	This study
WH1127	NCTC 12023; P22*attB*::*t*_L3_, P_tetA_ *gfp+*, *FRT*, *rgnB*; SGI1*attB*::*rgnB*, *FRT*, P_cat_ -10CAGCCA *tetR*, *t*_L3_	This study
SPL^c^ variant strains	NCTC 12023; P22*attB*::*t*_L3_, P_tetA_ *gfp+*, *FRT*, *rgnB*; SGI1*attB*::*rgnB*, *FRT*, P_cat_ -10CANNNN *tetR*, *t*_L3_	This study
WH1133	NCTC 12023; P22*attB*::*t*_L3_, P_tetA_ *gfp+*, *FRT*, *rgnB*; SGI1*attB*::*rgnB*, *FRT*, P_cat_ *tetR*, *t*_L3_; *trxA*-*TIP2*, *loxP*	This study
WH1134	NCTC 12023; P22*attB*::*t*_L3_, P_tetA_ *gfp+*, *FRT*, *rgnB*; SGI1*attB*::*rgnB*, *FRT*, P_cat_ -10CATTTA *tetR*, *t*_L3_;*trxA*-*TIP2*, *loxP*	This study
WH1135	NCTC 12023; P22*attB*::*t*_L3_, P_tetA_ *gfp+*, *FRT*, *rgnB*; SGI1*attB*::*rgnB*, *FRT*, P_cat_ -10CAGCCA *tetR*, *t*_L3_;*trxA*-*TIP2*, *loxP*	This study
WH1136	NCTC 12023; P22*attB*::*t*_L3_, P_tetA_ *gfp+*, *FRT*, *rgnB*	This study

a phage P22 attachment site within *thrW.*

b *Salmonella* Genomic Island 1, secondary attachment site located between *sodB* and *purR* [97].

c Synthetic Promoter Library.

**Table 2:** Plasmids used in this study.

Plasmid	Relevant characteristic(s)	Source or reference
pWH1012gfp+	Ap^R^ a, P_tetA_ *gfp+*, ori-ColE1	[52]
pWH2344	Ap^R^, Km^R^ b flanked by FRT sites, P_cat_ *tetR*, ori-R6Kγ	This study
pWH2352	Ap^R^, Km^R^ flanked by FRT sites, P_cat_ *tetR*, P_tetA_ *fluc*, ori-R6Kγ	This study
pWH2353	Ap^R^, *TIP2, lox66,* Cm^R^ c, *lox71*, ori-R6Kγ	This study
pWH2354	Cm^R^, *lacI^q^*, P_tac_ *trxA*-*TIP2*, ori-p15A	This study
pWH2358	Ap^R^, Km^R^ flanked by FRT sites, P_cat_ *tetR*, P_tetA_ *gfp+*, ori-R6Kγ	This study
pKD46	Ap^R^, Phage λ genes γ, β, *exo* under P_araB_ control, P_c_ *araC*, ori-R101	[53]
pCP20	Ap^R^, Cm^R^, *FLP^+^*, λ *c*I857^+^, λ *p*_R_ Rep^ts^	[98]
p2266	Ap^R^, Cre recombinase	Hammerschmidt W., unpublished data

a Ampicillin resistance.

b Kanamycin resistance.

c Chloramphenicol resistance.

### Construction of Plasmids and Reporter Strains

All *Salmonella* reporter strains generated are derived from NCTC 12023. Its published genome sequence [89] served as reference for designing the oligonucleotides used for recombination. The oligonucleotides used in this study are listed in Table S2 in Text S1 and were obtained from Eurofins MWG Operon. Restriction enzymes, ligase, Phusion and Taq polymerases were from New England BioLabs.

The reporter strains were established using the λ Red-mediated recombination technique [53], [90], [91]. Integration fragments carrying a kanamycin resistance cassette flanked by Flp recombinase target (FRT) sites were generated by polymerase chain reaction (PCR) using forward and reverse primers that introduced ∼100 bp long sequence elements for site-specific homologous recombination into the *Salmonella* genome. The PCR products were purified from agarose gels (NucleoSpin Gel and PCR Clean-up, Macherey-Nagel) and incubated with DpnI. To prepare strains for integration, cells were first transformed with the recombineering vector pKD46. An ampicillin-resistant transformant was inoculated 1∶100 from an LB overnight culture into SOB medium [88], supplemented with 10 mM L-arabinose and incubated at 28°C and 190 rpm. Cells were harvested at OD_600_ ∼ 0.6 and made electrocompetent following an established protocol [92]. Subsequently, the PCR product was electroporated and recombination candidates were selected on agar plates containing kanamycin. Correct integration was confirmed by PCR and sequencing following elimination of the resistance cassette by Flp-mediated recombination with the plasmid pCP20.

The strains WH1001 (P_tetA_ *fluc*), WH1102 (P_tetA_ *fluc*, P_cat_ *tetR*), WH1109 (P_tetA_ *fluc*, P_cat_ -10CATTTA *tetR*), as well as plasmids pWH2344, pWH2352, pWH2353 and pWH2354 (pTrx1-TIP2), are presented in detail in Buerger *et al.* (manuscript in preparation). Their construction is described in the Text S1 and the oligonucleotides, plasmids and strains used in their construction are listed in Table S3, Table S4, and Table S5 of Text S1, respectively.

The *gfp* reporter strain WH1104 was constructed by exchanging the *firefly luciferase* reporter gene located in the P22*attB* site of the strain WH1102 against *gfp+* [52]. The integration fragment containing *gfp+* under control of P_tetA_ was amplified from pWH2358 (the construction of this plasmid is described in detail in Text S1). The product to be integrated was generated by four consecutive PCR steps using the primer pairs P-A1 and P-A2 (for amplification of the *rgnB* terminator, the FRT-flanked kanamycin resistance, the P_tetA_ *gfp+* expression cassette and part of the *t*_L3_ terminator), P-B1 and P-B2 (for final amplification of *t*_L3_), P-C1 and P-C2 (for an overlap extension PCR with the first two products serving as templates) and P-D1 and P-D2 (for elongation of the homology arms). Homologous recombination was performed as described above, thereby deleting the P22*attB* site. After excision of the kanamycin resistance cassette, the strain was designated WH1104.

To introduce the P_cat_ -10CATTTA promoter variant into the strain WH1104, the PCR fragment used for creating strain WH1109 was re-amplified with the primers Int_for_rgnB30 and TP5. Upon its integration into the genome of WH1104, P_cat_ was exchanged against P_cat_ -10CATTTA. After deletion of the kanamycin resistance cassette, the resulting strain was called WH1106.

To obtain a *Salmonella gfp* reporter strain without Tet repressor, a fragment containing the P_tetA_ *gfp+* reporter element was amplified with the primer pair proA_WH1001_for and IS3_WH1001_rev using genomic DNA of the precursor strain of WH1104 as template. The fragment still carried the kanamycin resistance cassette present in the P_tetA_ *gfp+* element which was needed to select for the recombination event. Recombination into the NCTC 12023 genome was supported by stretches of ∼ 300 bp identical to the sequence surrounding the P22*attB* site at each end of the PCR product. After deletion of the kanamycin resistance cassette, the strain was called WH1136.

### Construction of the Synthetic Promoter Library

The *cat* promoter variant library was generated either by Combined Chain Reaction (CCR) [93] mutagenesis or by Two-Step PCR mutagenesis [94] using the degenerate oligonucleotide Pcat-10CANNNN (5′-GTTCCAACTTTCAC**CANNNN**GAAATAAGATCACTAC-3′). It carries the -10 element of P_cat_ (bold) with Ns at positions -11, -10, -9 and -8, with respect to the start site of transcription (underlined), for random mutagenesis and the adjacent nucleotides required for annealing of the primers. Because each nucleotide is randomly incorporated with identical probability at the positions marked with N, the pool should contain 4^4^ different oligonucleotides and, accordingly, 256 promoter variants.

For CCR, the integration fragment was first amplified from pWH2344 with the primers rgnB_term_for, lambda_term_rev and the 5′ phosphorylated mutagenesis primer Pcat-10CANNNN followed by re-amplification of the fragment with the primer pair tetR(B)408_rev and Int_for_rgnB30. For Two-Step PCR mutagenesis, a first PCR was performed with pWH2344 as template DNA and the primers Pcat-10CANNNN and tetR(B)408_rev. The resulting PCR product was used in a second PCR with the oligonucleotide Int_for_rgnB30 and pWH2344 serving again as template to synthesize the full-length integration cassette. In both approaches, the libraries consisted of P_cat_ -10CANNNN, a kanamycin resistance gene flanked by FRT sites, as well as sequences identical to *tetR* and the *rgnB* terminator. These were introduced for homologous recombination into the *gfp+* reporter strain WH1104, thereby replacing the wildtype *cat* promoter at the chromosomal level.

For further experiments, one strain was selected from the 360 promoter library candidates analyzed. It was designated WH1127 (P_cat_ -10CAGCCA) after deletion of the kanamycin resistance gene.

### Chromosomal Fusion of *TIP2* to *trxA*

Fusion of *TIP2* to the 3′ end of *trxA* was performed in strains WH1104, WH1106 and WH1127 by amplifying the *TIP2*-*lox66*-Cm^R^-*lox71* cassette from pWH2353 with the oligonucleotides fwd_1_int_P5 and rev_1_int_P5. A second primer pair, fwd_2_int_P5 and rev_2_int_P5, was used to extend the homology arms. The protocol employed for tagging *trxA* was identical to the recombineering protocol established to generate the reporter strains. After identification of positive integrants by PCR and sequencing, the resistance cassette was deleted by Cre recombinase expressed from plasmid p2266. This resulted in a single *loxP* site remaining as scar. The strains were named WH1133, WH1134 and WH1135, respectively.

### Luciferase Assay

Bacteria were inoculated in a 2.2 ml deep well plate (PeqLab) containing 1 ml LB with the appropriate antibiotics and incubated over night at 37°C and 800 rpm in a microplate shaker (TiMix 5 control, Bühler). The next day, 30 µl of the cell suspension were transferred into 1 ml fresh LB, supplemented with the necessary antibiotics, anhydrotetracycline (atc) or isopropyl-β-D-1-thiogalactopyranoside (IPTG). After the cells reached an OD_595_ ∼ 0.4, 300 µl of the cell suspension were transferred to a new deep well plate which contained 400 µl lysis buffer [25 mM potassium phosphate, pH 7.4, 2 mM EDTA, 5% (v/v) glycerol, 1% (v/v) Triton X-100, 2 mM dithiothreitol]. The cell suspension was frozen in liquid nitrogen and thawed at 37°C and 800 rpm. Meanwhile, the OD_595_ of the cultures was determined in a transparent 96 well flat bottom plate (Greiner) with a microplate reader (Infinite F200 Pro, TECAN). 100 µl of the thawed cell lysate were transferred to a white 96 well flat bottom plate (Greiner) and its luciferase activity determined in a microplate luminometer (Orion II, Berthold) by injecting 100 µl measurement buffer [100 mM potassium phosphate, pH 7.4, 5 mM ATP, 15 mM MgSO_4_, 0.25 mM D-Luciferin (P.J.K.)]. The resulting relative light units (RLU) were normalized to a 1 ml culture with OD_595_ = 1. Measurements were performed in triplicate (three colonies per strain) and at least three times.

### Measurement of GFP Fluorescence

Cells were grown over night in 1 ml LB medium in a 2.2 ml deep well plate (PeqLab) at 37°C and 800 rpm in a microplate shaker (TiMix 5 control, Bühler), with antibiotics if needed. The next day, 30 µl of the stationary phase cultures were reinoculated in 1 ml M9 minimal medium [88] supplemented with antibiotics, dox or IPTG if necessary and incubated just like the overnight cultures until OD_595_ ∼ 0.6. Cell growth was then stopped by incubating the plates on icewater for 10 min. Afterwards, 200 µl of the cell suspensions were transferred to a transparent 96 well flat bottom plate (Greiner) for measuring both the OD_595_ and the GFP fluorescence at 485 nm excitation and 535 nm emission in a microplate reader (Infinite F200 Pro, TECAN). Mean fluorescence values were normalized to a 1 ml culture with OD_595_ = 0.5. Measurements were carried out in duplicate (two colonies per strain) and at least three times.

### Western Blot Analysis

Strains were grown over night in LB with the required antibiotics at 37°C and 190 rpm. These cultures were diluted either 1∶100 in LB for *fluc*-carrying strains or 1∶33 in M9 minimal medium for strains carrying *gfp+*. Antibiotics were added if necessary. Cells were harvested at OD_600_ ∼ 0.6 and resuspended in 1×PBS [58 mM Na_2_HPO_4_, 17 mM NaH_2_PO_4,_ 68 mM NaCl]. The cells were lysed by sonication and the crude protein extracts separated from cell debris by centrifugation. The protein concentrations of the extracts were determined using the Bradford reagent (Bio-Rad). For SDS-PAGE, according to Schägger and von Jagow [95], 5–40 µg of the crude lysates were loaded on 15% (TetR) or 20% (Trx1-TIP2) gels and electrophoresis was carried out in a Mini-PROTEAN Tetra Cell (Bio-Rad). Afterwards, the gels were incubated for 30 min in 1×transfer buffer [192 mM glycine, 25 mM Tris, 3% (v/v) isopropanol, pH 8.9]. Meanwhile, a 0.45 µM polyvinylidene difluoride membrane (Roti-PVDF, Roth) was prepared by subsequent immersion in 100% methanol and 1×transfer buffer. Blotting of proteins to the membrane was carried out over night at 50 mA and 4°C in 1×transfer buffer in a Criterion Blotter (Bio-Rad). Next, the membranes were washed in 1×PBS-T (1×PBS supplemented with 0.1% (v/v) Triton X-100) and blocked in 5% skim milk solution (1×PBS-T, 5% (w/v) skim milk powder). The membranes were incubated with antibodies diluted in 2.5% skim milk solution for 1 h at room temperature. TetR was detected with a polyclonal rabbit antibody (SA-1851, lab stock) diluted 1∶5000. Endogenous Trx1 or Trx1-TIP2 fusions were detected using an anti-thio rabbit antibody (polyclonal, Sigma, dilution 1∶5000). Alternatively, a purified polyclonal anti-TIP2-rabbit antiserum (PINEDA, Germany) also allowed the detection of TIP2 fusion proteins (dilution 1∶200). DnaK served as loading control and was visualized with a monoclonal mouse antibody (clone 8E2/2, Biotrend, dilution 1∶10000). The secondary antibodies were horseradish peroxidase conjugated anti-rabbit (monoclonal, clone RG-96, Sigma, dilution 1∶5000) or anti-mouse (polyclonal, Sigma, dilution 1∶10000). For signal detection, membranes were incubated for 5 min with 1 ml ECL-solution (ECL Plus Kit, GE Healthcare) and exposed in a chemiluminescence imager (ChemiDoc XRS+ System, Bio-Rad). Analysis was carried out with ImageLab 3.0 (Bio-Rad).

### Sequence Analysis

For sequencing after plasmid construction and recombination, genomic (QIAamp DNA Mini Kit, Qiagen) or plasmid (NucleoSpin Plasmid, Macherey-Nagel) DNA was isolated. The respective region of interest was amplified and sequenced by GATC Biotech (Germany) using appropriate primers.

## Supporting Information

Figure S1 **Dose-response curve to analyze the sensitivity of TetR induction by dox in three promoter library mutants carrying identical -10 elements.** Controls were *Salmonella* WT and the strains containing P_tetA_ *gfp+* either with P_cat_ *tetR* or with P_cat_ -10CATTTA *tetR*. The control strains were incubated without and with 10 nM dox. The P_cat_ -10CATTTA mutant was also incubated with 400 nM dox for maximum induction of TetR. Bars illustrate the fluorescence intensity which was normalized to a 1 ml culture with OD_595_ = 0.5. The data are a representative set from at least three independent measurements and display the mean ± standard deviation.
(TIF)

Figure S2 **Repressed GFP fluorescence in the strains with the TetR-expressing promoters P_cat_, P_cat_ -10CATTTA or P_cat_ -10CAGCCA.** The promoter variants, as well as the control strains – *Salmonella* WT and the strain lacking TetR, leading to constitutive GFP expression (P_tetA_ *gfp+*) – were incubated without any inducer to display the activity of the P_tetA_ promoter when bound by TetR for comparing repression of reporter gene transcription in the strains with the P_cat_ variants driving TetR. The bars denote mean fluorescence values and are shown as counts per second at OD_600_ = 0.5. The data are a representative set from three independent measurements and display the mean ± standard deviation.
(TIF)

Figure S3 **Growth curves of the promoter variants P_cat_, P_cat_ -10CATTTA or P_cat_ -10CAGCCA.** The strains, including the *Salmonella* WT as reference, were cultivated in (A) LB-Lennox or (B) LPM (pH 5.8). The optical densities were determined at 600 nm and observed for 24 hours in LB-Lennox and for 32 hours in LPM. The data are a representative set from at least three independent measurements.
(TIF)

Text S1 **Supporting Material and Methods, Tables, and References.**
(DOC)
