# Supplementary figures and images for: Promoter Strength Driving TetR Determines the Regulatory Properties of Tet-Controlled Expression Systems
Source: PLoS One. 2012 Jul 27;7(7):e41620. doi: 10.1371/journal.pone.0041620 (PMC3407185; doi:10.1371/journal.pone.0041620)

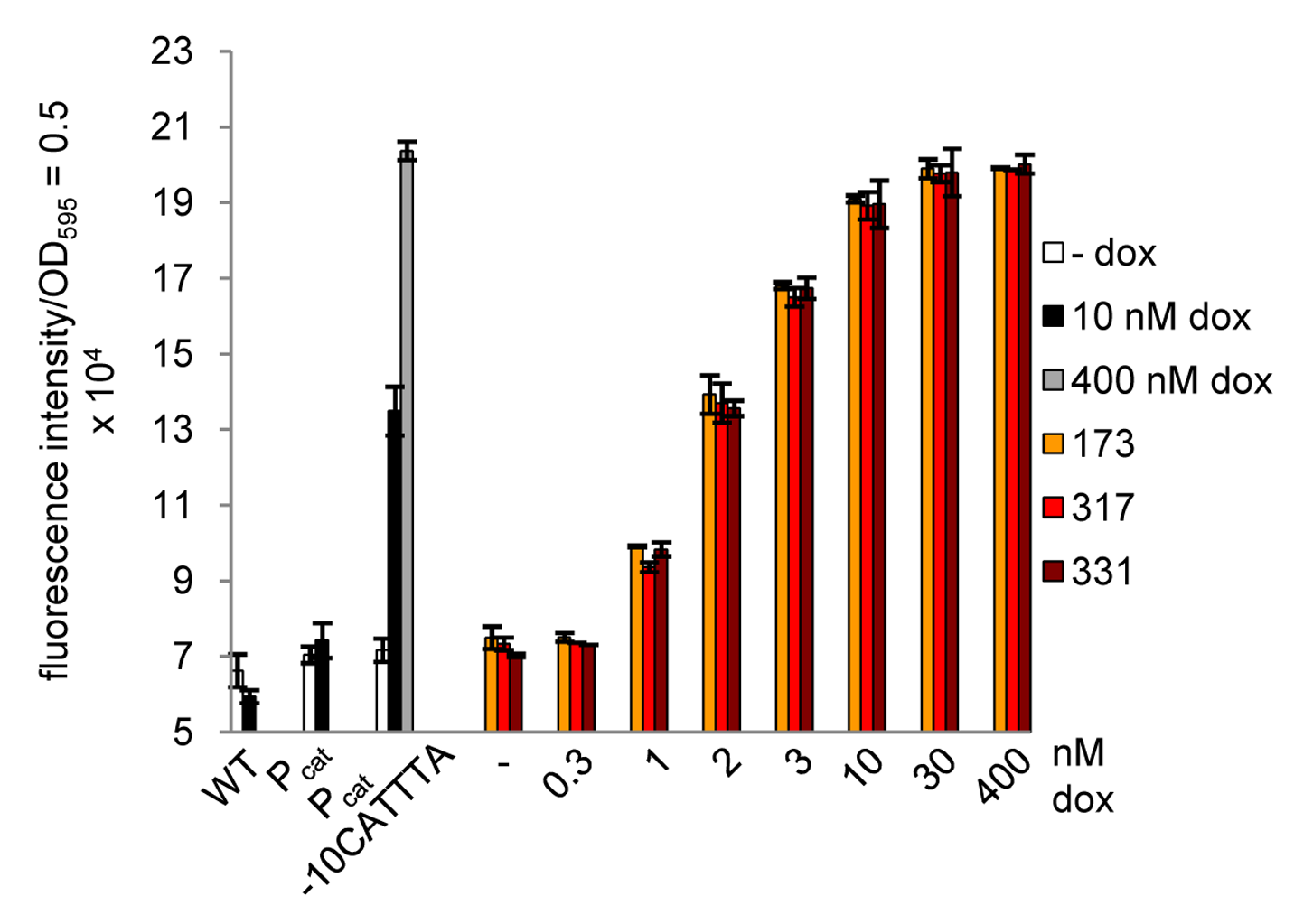

Supplement: Figure S1 — Dose-response curve to analyze the sensitivity of TetR induction by dox in three promoter library mutants carrying identical -10 elements. Controls were Salmonella WT and the strains containing PtetA gfp+ either with Pcat tetR or with Pcat -10CATTTA tetR. The control strains were incubated without and with 10 nM dox. The Pcat -10CATTTA mutant was also incubated with 400 nM dox for maximum induction of TetR. Bars illustrate the fluorescence intensity which was normalized to a 1 ml culture with OD595 = 0.5. The data are a representative set from at least three independent measurements and display the mean ± standard deviation. (TIF) [file pone.0041620.s001.tif]

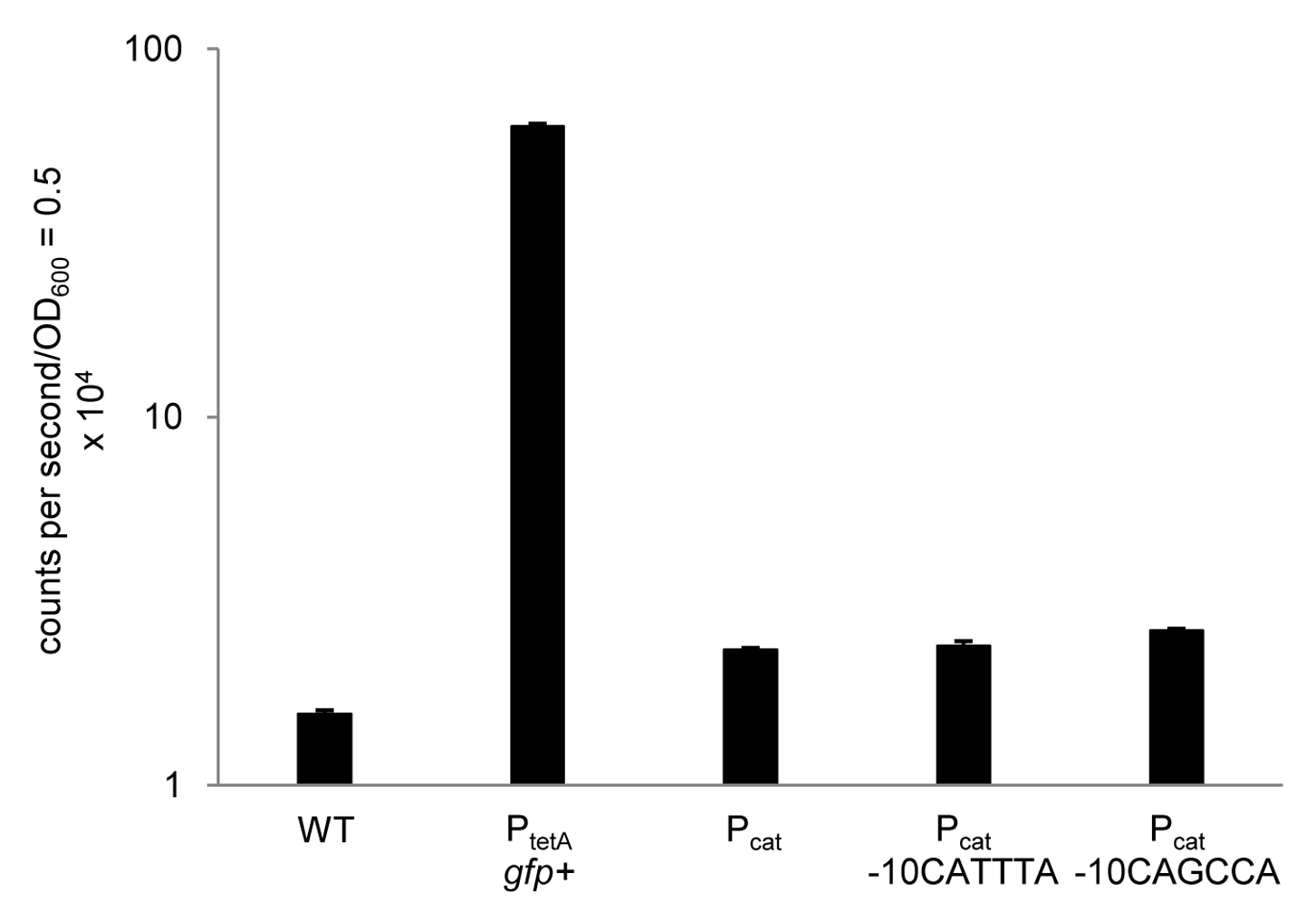

Supplement: Figure S2 — Repressed GFP fluorescence in the strains with the TetR-expressing promoters Pcat, Pcat -10CATTTA or Pcat -10CAGCCA. The promoter variants, as well as the control strains – Salmonella WT and the strain lacking TetR, leading to constitutive GFP expression (PtetA gfp+) – were incubated without any inducer to display the activity of the PtetA promoter when bound by TetR for comparing repression of reporter gene transcription in the strains with the Pcat variants driving TetR. The bars denote mean fluorescence values and are shown as counts per second at OD600 = 0.5. The data are a representative set from three independent measurements and display the mean ± standard deviation. (TIF) [file pone.0041620.s002.tif]

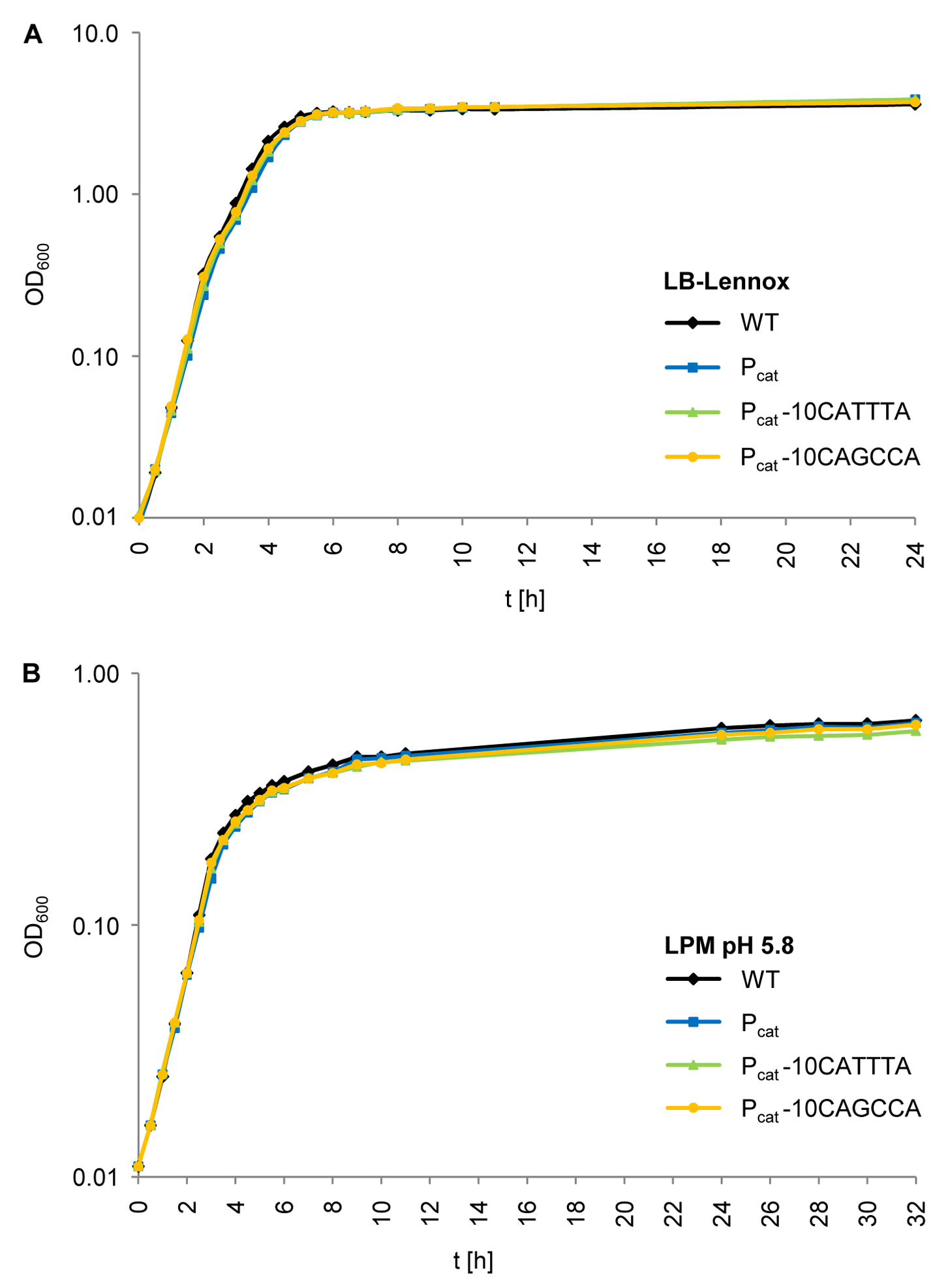

Supplement: Figure S3 — Growth curves of the promoter variants Pcat, Pcat -10CATTTA or Pcat -10CAGCCA. The strains, including the Salmonella WT as reference, were cultivated in (A) LB-Lennox or (B) LPM (pH 5.8). The optical densities were determined at 600 nm and observed for 24 hours in LB-Lennox and for 32 hours in LPM. The data are a representative set from at least three independent measurements. (TIF) [file pone.0041620.s003.tif]
