# Supplementary material for: Promoter Strength Driving TetR Determines the Regulatory Properties of Tet-Controlled Expression Systems
Source: PLoS One. 2012 Jul 27;7(7):e41620. doi: 10.1371/journal.pone.0041620 (PMC3407185; doi:10.1371/journal.pone.0041620)
Supplement: Text S1 — Supporting Material and Methods, Tables, and References. (DOC) [file pone.0041620.s004.doc]

# Supporting Information

Oligonucleotides, plasmids and strains used for construction of the plasmids and strains described in the Supporting Information are listed in Tables S3, S4 and S5, respectively.

# Supporting Material & Methods

# Construction of plasmid pWH2344

Cloning of pWH2344 was performed in the *E. coli* strain BW25141. It was generated by amplifying the P_cat_ *tetR*-encoding sequence from pWH2352 with the primer pair Pcat_SalI_for and tetR_BamHI_rev, followed by restriction of the PCR product with SalI and BamHI. It was ligated with likewise-treated pKD13.

# Construction of plasmid pWH2352

Cloning of pWH2352 was performed in the *E. coli* strain BW25141. It contains divergent, but independent expression cassettes for (i) constitutive expression of Tet repressor (TetR) by P_cat_ and (ii) a luciferase reporter gene under control of TetR (P_tetA_). First, the *tetR*-encoding sequence in pWH527 was isolated by restriction with NdeI and ApaI and ligated with likewise-digested pWH2077, which already contained a luciferase gene expressed by P_tetA_.

The vector pWH2077 was constructed to establish a regulatory system that could be integrated into a single-site of a bacterial genome. It contains independent, divergent expression cassettes for TetR (*tetR-F86A*; constitutively expressed) and a TetR-controlled luciferase reporter gene. The sequence encoding this reporter regulator setup was amplified with the primer pair 1371_for_SalI und 1371_rev_SalI from pWH2069 (construction available upon request). The PCR product and pKD13 were cut with SalI, and ligated after dephosphorylation of the vector.

# Construction of plasmid pWH2353

Cloning of pWH2353 was performed in the *E. coli* strain BW25141 to generate chromosomal *TIP2*-fusions to genes of interest. It was constructed via overlap extension PCR. First, the *TIP2*-encoding sequence was amplified by PCR from pWH403-P5-L9A with the primers FWD_P5-L9A_loxP66 and rev_P5-L9A_SalI. Next, a PCR fragment was generated with the primers Cm_for and rev_cat_lox66_P5-L9A and pWH2308 as template. It carries a chloramphenicol resistance cassette (Cm^R^) flanked by *lox* sites and a sequence identical to one end of the *TIP2*-containing PCR. Both fragments were added as templates to a third PCR with the primer pair Cm_for and rev_P5-L9A_SalI to generate the overlap fragment. This was cut with BspEI and SalI and ligated with pWH2308, restricted with the same enzymes.

The vector pWH2308 was cloned in the *E. coli* strain BW25141 and carries a chloramphenicol resistance cassette flanked by *lox* sites and located downstream of a sequence encoding *WTIP*. First, pWH2201-cat/loxP-WTIP (construction available upon request) was used as a template for amplification of the *lox* site-flanked resistance cassette and WTIP with the oligonucleotides Ctag_Spacer_for and Ctag_rev_ClaI. Restriction of this fragment with ClaI and SalI and ligation in likewise-restricted pKD13 followed.

# Construction of plasmid pWH2354

Cloning of pWH2354 was performed in the *E. coli* strain DH5α. To analyze induction of TetR by a plasmid-encoded Trx1-TIP2 fusion, pWH2354 was constructed by restriction of pWH1219 with RsrII and SpHI and ligation with a fragment containing the *trxA-TIP2* fusion present in pWH403-P5-L9A.

The vector pWH1219 with a Trx1-WTIP fusion under control of LacI was established by isolating the sequence encoding the Trx1-WTIP fusion from pWH2102 with the restriction enzymes SphI and EcoRI and ligation of this product with likewise-digested pWH794 (construction available upon request). This plasmid already carries an allele encoding *lacI*^q^.

## Construction of plasmid pWH2358

Cloning of pWH2358 was performed in the *E. coli* strain BW25141. This plasmid carries *gfp+* under control of a Tet-regulated promoter with a modified 5’-untranslated region and an adjacent *tetR* gene expressed by the *cat* promoter. The sequences upstream and downstream of the *gfp* start codon were altered by introducing a 33 bp sequence stretch including the Shine-Dalgarno (SD) sequence from the ribosomal protection protein TetO upstream of the ATG startcodon, as well as the codons of its first four amino acids following the start codon. Since TetO has a very efficient consensus SD sequence, this modification resulted in increased reporter protein expression (J. Buerger, unpublished data). In the first PCR, the P_tetA_ fragment was amplified from pWH2352. While TP5 served as forward primer, the modified *gfp+* upstream region was introduced by the reverse primer Rev_Ptet_mod_wt_TetOup. This fragment contained 19 bp upstream of the SD, the SD sequence itself (AGGAGG), followed by 8 bp serving as spacer to the ATG start codon. In the second PCR, *gfp+* was amplified together with the first four codons of the *tetO* gene from pWH1012gfp+ using the primer pair Fwd_Ptet_mod_tetO_up_wt and Rev_gfp+_aatII. Then, an overlap extension PCR of the two products using the primers TP5 and Rev_gfp+_aatII was performed. After restriction of both PCR product and vector pWH2352 with ApaI and AatII, their subsequent ligation followed, joining *gfp+* with the modified upstream sequence.

# Construction of strain WH1001

WH1001 was established from WH999. WH999 was created by amplifying a P_tetA_ containing sequence with an FRT-flanked kanamycin resistance cassette from pWH411 (construction available upon request) with tetK_int_for and tetK_int_rev. An additional second PCR with tetK_int_for2 and tetK_int_rev2 elongated the sequence identity to the *rgnB* and *t*_L3_ terminators present in WH945. Electroporation of this strain with the amplification product and deletion of the resistance cassette led to WH999. WH1001 was finally established by amplifying a fragment containing *fluc* and a kanamycin resistance cassette flanked by FRT sites with Fluc_int_for and Fluc_int_rev from pWH1995 (construction available upon request). Next, the primer pairs Fluc_int_for2, Fluc_int_rev2, Fluc_int_for3 and Fluc_int_rev3 were used in subsequent PCRs to lengthen the region of sequence identity to WH999 step by step. Afterwards, the resulting fragment was electroporated into this strain.

The starting strain for all reporter strains was WH945, which carries *rgnB* and *t*_L3_ terminators that establish a transcriptionally insulated site in the genome which can serve as an integration site for different expression systems. The primer pairs P22_ for and P22_ rev and P22_int_for2 and P22_int_rev2 were used to amplify and elongate a kanamycin resistance cassette flanked by terminators present in pKD13. After electroporation of this latter PCR fragment in NCTC 12023 and deletion of the resistance cassette, the resulting strain was named WH945.

## Construction of strain WH1102

WH1102 was created by amplifying a cassette from pWH2344, containing *tetR* constitutively expressed by P_cat_ and a kanamycin resistance cassette flanked by FRT sites, with the oligonucleotides int_f1_neu and int_r1_neu to create sequence identity to the secondary *attB* site of *Salmonella* genomic island 1 (SGI1), located between *sodB* and *purR* . The region of sequence identity to SGI1*attB* was elongated twice by PCR with the oligonucleotide pairs PcatTetRB_int_f2, PcatTetRB_int_r2 and PcatTetRB_int_f3, PcatTetRB_int_r3. This PCR fragment was then electroporated into WH1001. After deletion of the resistance cassette, the resulting strain was named WH1102.

# Construction of strain WH1109

To generate a reporter strain with a lower steady-level of TetR, P_cat_ was amplified from pWH2344 with the mutagenesis primer Pcat-10TTA_for and TP5. The resulting PCR product was used in a Two-Step PCR with the oligonucleotide Int_for_rgnB30 to amplify a kanamycin resistance cassette flanked by FRT sites and pWH2344 serving as template. After an additional amplification with the primers Int_for_rgnB30 and TP5 to extend the homologous sequence, we integrated this product into WH1102, resulting in the reporter strain WH1109 after excision of the resistance cassette.

## Growth analysis of the three *gfp* reporter strains with P_cat_, P_cat_ -10CATTTA and P_cat_ -10CAGCCA driving TetR expression

Growth behavior was analyzed in LB-Lennox rich medium [5 g/l NaCl, 5 g/l yeast extract, 10 g/l Bacto-tryptone] or in LPM minimal medium (pH 5.8) . Cells were grown over night in 20 ml LB-Lennox at 37 °C and 190 rpm. The next day, the cell suspensions were transferred to a start OD_600_ of 0.01 either into 100 ml fresh LB-Lennox or 100 ml LPM and incubated at 37 °C and 180 rpm. For incubation in LPM, over night cultures were centrifuged, washed and resuspended in 20 ml LPM prior to inoculating the fresh medium. The densities of the cultures were monitored for 24 hours in LB-Lennox and for 32 hours in LPM, first every 30 minutes for six or seven hours, then every hour until incubation for a total of 11 hours. The next day, a final measurement followed after 24 hours for cultivation in LB-Lennox or, for cultivation in LPM, after several measurements every two hours up to the 32 hour time point. The measurements were carried out in duplicate at least three times.

## Measurement of repressed GFP fluorescence in the promoter variants P_cat_, P_cat_ -10CATTTA and P_cat_ -10CAGCCA

Cells were grown over night in 4 ml LB medium at 37 °C and 200 rpm. The next day, 200 µl of the stationary phase cultures were reinoculated in 8 ml M9 minimal medium and incubated just like the overnight cultures until an OD_600_ ~ 0.6. Cell growth was then stopped by incubating the cultures on ice for 10 min. Afterwards, the cultures were centrifuged, washed in 8 ml 1× PBS [58 mM Na_2_HPO_4_, 17 mM NaH_2_PO_4_, 68 mM NaCl], centrifuged again, and resuspended in 2 ml 1× PBS. A 1:10 dilution of the suspension was prepared to photometrically determine cell densities at 600 nm. The cultures were then adjusted with 1× PBS to a final OD_600_ of 0.5 in a total volume of 2 ml for measuring GFP fluorescence in a Spex Fluorolog 1680 (ISA, Edison, NJ, USA, slit width: 1.8 mm), equipped with two monochromators (excitation wavelength: 491 nm; emission wavelength: 512 nm). Measurements were carried out three times in duplicate (two colonies per strain).

## P_cat_ -10 element sequences of the seven most sensitive promoter variants

**Table S1.** P_cat_ -10 element sequences of the seven most sensitive promoter variants.

| **promoter variant clone #** | **P_cat_ -10 element sequence** |
| --- | --- |
| 173 | CAGCCA |
| 210 | CATCCA |
| 221 | CACTGA |
| 317 | CAGCCA |
| 318 | CAGGCG |
| 331 | CAGCCA |
| 369 | CACGGA |

## Oligonucleotides used in this study

**Table S2.** Oligonucleotides used in this study.

| **Designation** | **Sequence 5’- 3’** |
| --- | --- |
| Rev_Ptet_mod_wt_TetOup | GTAATTTTCCTCCTATCAACACCCAAAAAAGGGCTCTAGAAGCTTCTGTCTC |
| Fwd_Ptet_mod_tetO_up_wt | GTGTTGATAGGAGGAAAATTACATGAAAATAATTAACGCTAGCAAAGGAGAAG |
| Rev_gfp+_aatII | TGATGACGTCTTATTTGTAGAGCTCATCC |
| P-A1 | CGTGATTCACCCGCGTGAACACACCCTTCTCAGGGCCGATATAGCTCAGTTGGTAGAGCAGCGCGATGGTAGTGTGGGGTCTCC |
| P-A2 | CGCCATTAATTCACTGATCAGTGATAAGCTGTCAAACATGAGAATTAGCTTAAGTAGGTAAAAATATAATGACCCTCTTGATAAC |
| P-B1 | TAATTCTCATGTTTGACAGCTTATC |
| P-B2 | CTTCGTCTGTTTCTACTGGTATTG |
| P-C1 | ATAGCCGGTTGAGTAGCAAGGCTATTGACGCACCGCCCGGTTAGTTTTAACCTTCTACCCCGTGATTCACCCGCGTGAAC |
| P-C2 | CCCGAGAGCAAAAAATGGTGTTTTTGAGAAATGAGGTTGTACATAAGTGATTGATTTAGACTTCGTCTGTTTCTACTGGTATTG |
| P-D1 | GCTTTGGCGATGGTACGATTCGTGCGTAATTAATGACGAGCGGTGCAAAAATAGCCGGTTGAGTAGCAAGG |
| P-D2 | GTTAGGTGGGATACTGCTTATGTTTTGCTAGTTGTGTACCCGAGAGTGTACCCGAGAGCAAAAAATGG |
| TP5 | GTGCCTATCTAACATCTCAA |
| Int_for_rgnB30 | CGCCATAAACTGCCAGGCATCAAATTAAGC |
| proA_WH1001_for | CGTGAACGAAGTGGATTCCGCTGCGGTTTATG |
| IS3_WH1001_rev | AGAACCTCGCTTAGGGTTGTGCCCATATTACG |
| rgnB_term_for | GATGGTAGTGTGGGGTCTCC |
| lambda_term_rev | CTTCGTCTGTTTCTACTGGTATTG |
| Pcat-10CANNNN | GTTCCAACTTTCACCANNNNGAAATAAGATCACTAC |
| tetR(B)408_rev | AGCGCTGAGTGCATATAACG |
| fwd_1_int_P5 | GTCAGTTGAAAGAGTTTCTCGACGCCAATCTGGCGTCGGGTGGAGCTGATGATTC |
| rev_1_int_P5 | TACAGCGCCTTTGTCATTCGACGTATAAAAGGTAATAACTTCGTATAATGTATGC |
| fwd_2_int_P5 | CGGCAACCAAAGTAGGCGCACTGTCTAAAGGTCAGTTGAAAGAGTTTCTC |
| rev_2_int_P5 | ACCTTAATCATTCGTCGAATGACAGACGCCTGACCATACAGCGCCTTTGTCATTCG |

## Supplemental Oligonucleotides

**Table S3.** Supplemental oligonucleotides.

| **Designation** | **Sequence 5’- 3’** |
| --- | --- |
| Pcat_SalI_for | GCAAGTCGACAATCCTTAAGCGATCCGCGATGCC |
| tetR_BamHI_rev | GCCGGGATCCGTTAAGACCCACTTTCACATTTAAG |
| 1371_for_SalI | TAGTATGTCGACCTAGGTTAAGACCCACTTTCAC |
| 1371_rev_SalI | TGTTACGTCGACCTGACGTCTTACAATTTGGAC |
| FWD_P5-L9A_loxP66 | GTATGCTATACGAAGTTATGTTACCAATGCCACATCCACATC |
| rev_P5-L9A_SalI | CTATGTCGACTCGGGTGGAGCTGATGATTCG |
| Cm_for | CGCCCCGCCCTGCCACTC |
| rev_cat_lox66_P5-L9A | CATTGGTAACATAACTTCGTATAGCATAC |
| Ctag_Spacer_for | TAGTGTGTCGACTCGGGTGGAGCTTGGTGGAC |
| Ctag_rev_ClaI | TGTCGATCGATTGGTCATGCATAAC |
| tetK_int_for | ATTACACGTCTTGAGCGATTGTGTAGGCTGGAGCTGCTTCTTCGAAGTTCCTATACTTTC |
| tetK_int_rev | CAAACATGAGAATTAATTCCGGGGATCCGTCGACCTGCAGATCGTGAGGATGCGTCATAG |
| tetK_int_for2 | CCTTTTTGCGTGGCCAGTGCCAAGCTTGCTGCAGATTGCAGCATTACACGTCTTGAGCG |
| tetK_int_rev2 | TGCGTCATCGCCATTAATTCACTGATCAGTGATAAGCTGTCAAACATGAGAATTAATTCC |
| Fluc_int_for | TCTCCGCCTGCAGGCTTACTAACAAACAAGGAGGAAAGAACAATGGAAGACGCCAAAAAC |
| Fluc_int_rev | GTCAAACATGAGAATTAATTCCGGGGATCCGTCGCGATTGTGTAGGCTGGAGCTGCTTCG |
| Fluc_int_for2 | AAAAGTGAAATGAATAGTTCGGATCTTCCAACCTACCAGTTCTCCGCCTGCAGGCTTAC |
| Fluc_int_rev2 | ATGCGTCATCGCCATTAATTCACTGATCAGTGATAAGCTGTCAAACATGAGAATTAATTC |
| Fluc_int_for3 | ATCATTGATAGAGTTATTTTACCTCTCCCTATCAGTGATAGAGAAAAGTGAAATGAATAG |
| Fluc_int_rev3 | TTAATTCCGGGGATCCGTCGACCTGCAGTTATCGTGAGGATGCGTCATCGCCATTAATTC |
| P22_ for | GCTATTGACGCACCGCCCGGTTAGTTTTAACCTTCTACCCACCTGCATCGATGGCCCCCC |
| P22_ rev | ATCGGCCCTGAGAAGGGTGTGTTCACGCGGGTGAATCACGCAATCCAGTGCAAAGCTAGC |
| P22_int_for2 | TAATGACGAGCGGTGCAAAAATAGCCGGTTGAGTAGCAAGGCTATTGACGCACCGCCCGG |
| P22_int_rev2 | ACCTTCGCATTACGAATGCGCTGCTCTACCAACTGAGCTATATCGGCCCTGAGAAGGGTC |
| int_f1_neu | CATTCCCGCACAAAAATCTCTCATGGTGCCACCTGCATCGATGG |
| int_r1_neu | GGAAAAGGGGAAGCGTTAAAAACAGGCAAAGCCTCGCAATCCAGTG |
| PcatTetRB_int_f2 | CCTTTTTATTCCCACACAAAAAGTGATATAGCGCATTTTTGCACATTCCCGCACAAAAATCTCTCATGG |
| PcatTetRB_int_r2 | GCCGTTACGCTAATTCAGTAAGCCTGGAATTTTGTCTGATCTTGAGGAAAAGGGGAAGCGTTAAAAACAG |
| PcatTetRB_int_f3 | GAAAATTTCAGGTCGGTACTCATCAAGTTTTCTCCTTTTTATTCCCACACAAAAAGTGATATAG |
| PcatTetRB_int_r3 | CGACACGGAACGTCAGGAAGGGGTCAATGGCCGTTACGCTAATTCAGTAAGCCTGGAATTTTG |
| Pcat-10TTA_for | CAACTTTCACCATTTAGAAATAAGATCACTAC |

# Supplemental plasmids

**Table S4.** Supplemental plasmids.

| **Plasmid** | **Relevant characteristic(s)** | **Source or reference** |
| --- | --- | --- |
| pKD13 | Ap^R^, Km^R^ flanked by FRT sites, ori-R6Kγ |  |
| pWH527 | Km^R^, *tetR*, *lacI^q^*, ori-p15A |  |
| pWH2077 | Ap^R^, Km^R^ flanked by FRT sites, P_cat_ *tetR-F86A*, P_tetA_ *fluc*, ori-R6Kγ | This study |
| pWH403-P5-L9A | Ap^R^, P_tac_ *trxA-TIP2*, ori-pMB1 |  |
| pWH2308 | Ap^R^, *WTIP, lox66,* Cm^R^*, lox71*, ori-R6Kγ | This study |
| pWH1219 | Cm^R^, *lacI^q^*, P_tac_ *trxA*-*WTIP*, ori-p15A | This study |
| pWH2102 | Ap^R^, *lacI^q^*, P_tac_ *trxA-WTIP*, ori-pMB1 |  |
| pWH794 | Cm^R^, *lacI^q^*, *tetR-F86A*, ori-p15A | This study |
| pWH411 | Ap^R^, Km^R^, flanked by FRT sites, *t*_L3_, P_tetA_, *rgnB*, ori-R6Kγ | This study |
| pWH1995 | Ap^R^, *tetR*, ori-pMB1 | This study |

## Supplemental strains

**Table S5.** Supplemental strains.

| **Strain** | **Relevant characteristic(s)** | **Source or reference** |
| --- | --- | --- |
| *E. coli* K-12 DH5α | *recA*1, *endA*1, *gyrA*96, *thi*, *relA*1, *hsd*R17 (r_K_^-^, m_K_^+^), *supE*22, Φ 80d*lacZ*ΔM15, Δ*lac*U169 |  |
| WH999 | NCTC 12023; P22*attB*::*rgnB*, *FRT*, P_tetA_, *t*_L3_ | This study |
| WH945 | NCTC 12023; P22*attB*::*rgnB*, *FRT*, *t*_L3_ | This study |

# Supporting References

1. Goeke D, Kaspar D, Stoeckle C, Grubmüller S, Berens C, *et al.* (2012) Short peptides act as inducers, anti-inducers and corepressors of Tet repressor. J Mol Biol 416: 33-45.

2. Klotzsche M, Goeke D, Berens C, Hillen W (2007) Efficient and exclusive induction of Tet repressor by the oligopeptide Tip results from co-variation of their interaction site. Nucleic Acids Res 35: 3945-3952.

3. Sougakoff W, Papadopoulou B, Nordmann P, Courvalin P (1987) Nucleotide-Sequence and distribution of gene *tetO* encoding tetracycline resistance in *Campylobacter coli*. FEMS Microbiol Lett 44: 153-159.

4. Doublet B, Golding GR, Mulvey MR, Cloeckaert A (2008) Secondary chromosomal attachment site and tandem integration of the mobilizable *Salmonella* genomic island 1. PLoS One 3: e2060.

5. Kammann M, Laufs J, Schell J, Gronenborn B (1989) Rapid insertional mutagenesis of DNA by polymerase chain reaction (PCR). Nucleic Acids Res 17: 5404.

6. Coombes BK, Brown NF, Valdez Y, Brumell JH, Finlay BB (2004) Expression and secretion of *Salmonella* pathogenicity island-2 virulence genes in response to acidification exhibit differential requirements of a functional type III secretion apparatus and SsaL. J Biol Chem 279: 49804-49815.

7. Sambrook J, Russell DW (2001) Molecular Cloning. Cold Spring Harbor, New York: Cold Spring Harbor Laboratory Press.

8. Datsenko KA, Wanner BL (2000) One-step inactivation of chromosomal genes in *Escherichia coli* K-12 using PCR products. Proc Natl Acad Sci USA 97: 6640-6645.

9. Klotzsche M, Berens C, Hillen W (2005) A peptide triggers allostery in Tet repressor by binding to a unique site. J Biol Chem 280: 24591-24599.

10. (1986) BRL pUC host: *E. coli* DH5α competent cells. Focus 8: 9.
